# Tachykinin-related peptides modulate immune-gene expression in the mealworm beetle *Tenebrio molitor* L.

**DOI:** 10.1038/s41598-022-21605-6

**Published:** 2022-10-14

**Authors:** Arkadiusz Urbański, Paul Johnston, Elisa Bittermann, Maryam Keshavarz, Véronique Paris, Karolina Walkowiak-Nowicka, Natalia Konopińska, Paweł Marciniak, Jens Rolff

**Affiliations:** 1 Department of Animal Physiology and Developmental Biology, Adam Mickiewicz University, Poznań, Poland; 2 Evolutionary Biology, Institute for Biology, Freie Universität Berlin, Berlin, Germany; 3 Berlin Centre for Genomics in Biodiversity Research, Berlin, Germany; 4 Leibniz-Institute of Freshwater Ecology and Inland Fisheries (IGB), Berlin, Germany; 5 Bio 21 Institute, University of Melbourne, Parkville, VIC 3052 Australia; 6 Berlin-Brandenburg Institute of Advanced Biodiversity Research (BBIB), Berlin, Germany

**Keywords:** Physiology, Immunology, Antimicrobial responses, Innate immunity, Environmental social sciences, Environmental economics, Transcriptomics

## Abstract

Tachykinins (TKs) are a group of conserved neuropeptides. In insects, tachykinin-related peptides (TRPs) are important modulators of several functions such as nociception and lipid metabolism. Recently, it has become clear that TRPs also play a role in regulating the insect immune system. Here, we report a transcriptomic analysis of changes in the expression levels of immune-related genes in the storage pest *Tenebrio molitor* after treatment with Tenmo-TRP-7. We tested two concentrations (10^–8^ and 10^–6^ M) at two time points, 6 and 24 h post-injection. We found significant changes in the transcript levels of a wide spectrum of immune-related genes. Some changes were observed 6 h after the injection of Tenmo-TRP-7, especially in relation to its putative anti-apoptotic action. Interestingly, 24 h after the injection of 10^–8^ M Tenmo-TRP-7, most changes were related to the regulation of the cellular response. Applying 10^–6^ M Tenmo-TRP-7 resulted in the downregulation of genes associated with humoral responses. Injecting Tenmo-TRP-7 did not affect beetle survival but led to a reduction in haemolymph lysozyme-like antibacterial activity, consistent with the transcriptomic data. The results confirmed the immunomodulatory role of TRP and shed new light on the functional homology between TRPs and TKs.

## Introduction

Tachykinins (TKs) are one of the largest neuropeptide families that is conserved across the animal kingdom, from Cnidaria to vertebrates^1^. In insects, neuropeptides with similar structural properties are classified as tachykinin-related peptides (TRPs). TRPs, like TKs, participate in the regulation of many processes. TRPs can, *inter alia*, modulate the contractile activity of visceral muscles, nociception, and lipid metabolism^1,2^. Recent results also indicate that TRPs are a very important part of the hormonal axis, which is crucial for fast reactions by insects to stress conditions^2–4^. This importance is indicated by the close interplay between TRPs, insulin-like peptides (ILPs) and adipokinetic hormones (AKHs), a functional homologue of vertebrate glucagon. The regulatory role of these hormones is mostly based on the adjustment of insect metabolism to the current physiological state and to the direct or indirect regulation of insect immune system activity^3–8^. Despite the well-known immunotropic properties of AKHs and ILPs, our knowledge about the role of TRPs in the activity of different immune mechanisms is very limited.

Our previous research provided the first evidence for the possible role of TRPs in regulating the insect immune system in the mealworm beetle *Tenebrio molitor*^9^, a storage pest species. The application of Tenmo-TRP-7 (one of the TRPs identified in *T. molitor*) elicited many physiological effects, resulting in an increase in the total number of circulating haemocytes, a decrease in the number of phagocytic immune cells, and altered haemocyte adhesion. Moreover, Tenmo-TRP-7 enhanced the activity of phenoloxidase (PO) in *T. molitor* haemolymph^9^, which is one of the main components of the insect immune system^10^. The injection of Tenmo-TRP-7 also reduced the DNA damage observed in haemocytes^9^. We also identified and predicted the sequence and structure of the TRP receptor (TRPR) and confirmed the presence of gene expression encoding TRPR in *Tenebrio* haemocytes. This result supports the notion that TRPs directly influence the activity of insect haemocytes^9^. In addition, research conducted by Kamareddine et al.^11^ showed that the innate immune deficiency (IMD) pathway can regulate TRP transcription in the *Drosophila melanogaster* gut. However, the regulatory mechanisms of TRPs are still unknown. Based on research conducted in other model organisms, especially vertebrates, one can hypothesize that the application of TRPs could induce changes in the expression level of a wide spectrum of immune-related genes. Current research clearly indicates that in vertebrates, the application of substance P (SP, one of the vertebrate TKs) affects the expression level of genes encoding various cytokines^12^. Here, we report the transcriptomic changes in immune-related tissues (fat body and haemocytes) of *T. molitor* after Tenmo-TRP-7 application using RNA-seq. In addition, we investigated insect survival after applying TRP, and we tracked the lysozyme-like antimicrobial activities of *T. molitor* haemolymph.

## Materials and methods

### Insects

To control for age- and sex-specific differences in immune system functioning, only 7-day-old adult males of *T. molitor* were used. The beetles were reared at the Institute of Zoology, Freie Universität Berlin and at the Department of Animal Physiology and Developmental Biology, Adam Mickiewicz University in Poznań according to a method described by El-Shazely et al.^13^ and Urbański et al.^9^. Adult males were kept in an incubator under stable conditions (dark, 28 °C). Beetles were kept in sterile, compartmentalized square plastic dishes with oatmeal and apple pieces.

### Neuropeptide and tissue collections

Similar to previous research, the neuropeptide Tenmo-TRP-7 (MPRQSGFFGMRa) was used for all the experiments^9^. Tenmo-TRP-7 was synthesized by Creative Peptides (Shirley, NY, USA; purity > 95% HPLC). Tenmo-TRP-7 was used because of its structural similarity to SP, which possesses immunomodulatory activity in vertebrates^6,9^.

The neuropeptide solution in physiological saline (2 μL; 274 mM NaCl, 19 mM KCl, 9 mM CaCl_2_) was injected under the coxa of the third pair of legs 6 or 24 h before tissue collection. In the experiment, two concentrations of Tenmo-TRP-7 were used, 10^–7^ and 10^–5^ M (for final concentrations in the *Tenebrio* haemocoel of 10^–8^ and 10^–6^ M, respectively)^9,14^. In the “Results and discussion” section, the results are related to the final concentration of Tenmo-TRP-7 in the insect haemocoel. The neuropeptide concentrations are based on previous research on TRPs in the *Tenebrio* immune system activity and on other studies in insects^9,15,16^.

Before neuropeptide injection or haemolymph and fat body collection, the beetles were anaesthetized with CO_2_. Haemolymph samples (depending on the experiment, 2 or 5 µL) were collected by cutting the tibia of the first pair of legs. The fat body was collected under sterile conditions just after beetle decapitation using microsurgical tools and a dissecting microscope (Zeiss Stemi 508, Carl Zeiss, Jena, Germany). For the transcriptomic analyses, the fat body and haemolymph were pooled. The collected samples were placed directly in RNA Lysis buffer (Zymo, Irvine, USA). For each experimental condition, at least three biological replicates were collected. One biological replicate contained tissues pooled from 5 individuals. Haemolymph samples were also used for spectrophotometric analysis of their lysozyme-like activity.

### Survival

The survival study was modified according to the method described previously by El-Shazely et al.^13^. Ten male *T. molitor* individuals that were injected with physiological saline or a Tenmo-TRP-7 solution at concentrations of 10^–7^ and 10^–5^ M were kept in a plastic box for 21 days. This box of 10 was considered one biological replicate. The number of individuals was checked every day at the time of the experiment. Each research variant (control, 10^–8^ and 10^–6^ M) was repeated at least 5 times (5 replications × 10 individuals = 50 individuals per treatment).

### Sequencing, transcriptome assembly and analysis

We used RNA-seq to study the expression of immune-related genes in *T. molitor* after neuropeptide administration. The fat body and haemolymph were suspended in RNA Lysis buffer (Zymo Research, Irvine, USA) and homogenized using TissueLyser II (Qiagen, Hilden, Germany). RNA isolation was performed using the Zymo Quick RNA MiniPrep kit according to the manufacturer’s protocol, including sample incubation with DNase (Zymo Research, Irvine, USA). The quantity and quality of the RNA were determined with a NanoDrop (Thermo Fisher Scientific, Waltham, USA) and BIOANALYZER 2100 (Agilent, Santa Clara, USA). The mRNA library was prepared using a NEXTflexTM Rapid Directional mRNA-seq Kit (Bio Scientific, Austin, USA). To sequence the prepared library, the Illumina NextSeq500/550 platform was used (Illumina, San Diego, USA).

The raw data processing was based on methods described by Johnston et al.^17^ and He et al.^18^. First, Trimmomatic, part of Trinity (v. 2.2.0), was used for data trimming and filtering. During this step, barcodes, adapters, short reads (< 25 bp) and reads of low quality were removed. Trinity was used to assemble pair-end reads. The quality of the assembly was assessed by BUSCO v. 2 with the Arthropod BUSCO set from OrthoDB (version 9). The transcriptome was annotated in accordance with the Trinotate annotation suite guidelines. Trimmed reads were mapped to the reference assembly using RSEM and Bowtie. The difference in gene expression was analysed using the R Bioconductor package DESeq. Transcripts with a minimum of fourfold change in expression at *p* ≤ 0.05 were extracted and clustered using the R package DIRECT^17,19^. GO PANTHER (http://pantherdb.org) was used for Gene Ontology (GO) analyses. Based on the resulting transcriptomic data, GO term enrichment analyses on different sets of differentially expressed genes were performed using Goseq^20^. Further analysis was conducted based on the method described by Bonnot et al.^21^. The identification of the most representative GO terms from the list of enriched terms using REVIGO (http://revigo.irb.hr) was performed^22^. The lists of GO terms were prepared by applying a stringent dispensability cut-off (< 0.05). For the graphical presentation of the obtained data, *ggplot2* (https://ggplot2.tidyverse.org) for RStudio was used (http://www.rstudio.com)^21,23,24^. The GO enrichment analyses for GO terms classified as “cellular components” are presented in the Supplementary Materials (Figs. S3–S6). The analysis was performed at the Institute of Biology, Freie Universität Berlin and Berlin Centre for Genomics in Biodiversity Research (BeGenDiv).

### Expression level of selected immune-related genes-quantitative PCR assay

The transcriptomic data were verified by analysing the expression levels of selected immune-related genes. Immune-related tissues (fat body and haemocytes) were transferred to 200 μL of RNA lysis buffer (Zymo Research, Irvine, USA) and homogenized for 2 min using a pellet homogenizer (Kimble Chase, USA). For each biological replicate, the tissues collected from 5 individuals were pooled. The homogenized tissues were immediately frozen in liquid nitrogen and stored at − 80 °C. For RNA isolation, a Quick-RNA^®^ Mini-Prep kit (Zymo Research, Irvine, USA) was used. After RNA isolation, DNase treatment of samples with a Turbo DNase Kit (Thermo-Fisher Scientific, Waltham, USA) was performed. Quantification and verification of isolated RNA were performed using a Synergy H1 Hybrid Multi-Mode Microplate Reader (BioTek, Winooski, USA). Reverse transcription of the same amount of isolated RNA (200 ng) to cDNA was accomplished using the RevertAid^®^ First Strand cDNA Synthesis Kit (Thermo-Fisher Scientific, Waltham, USA) according to the manufacturer’s protocol.

The primers for PCR were based on primer sequences previously published by Jacobs et al.^25^ (Supplementary Materials, Table S1) and were synthesized by Institute of Biochemistry and Biophysics of Polish Academy of Science in Warsaw. Based on the transcriptomic data, genes encoding attacin 2, tenecin 3 and the Toll receptor were selected for the analysis. Reverse transcription quantitative PCR (RT-qPCR) was performed on a Corbett Research RG-6000 Real-Time PCR Thermocycler (Qiagen, Hilden, Germany) with Fast SYBR Green Master Mix (Applied Biosystems, Thermo Fisher Scientific, Waltham, USA) according to the manufacturer’s protocol. The expression level of the gene encoding *T. molitor* ribosomal protein L13a (TmRpL13a) was used as an internal control to normalize differences in template concentrations between samples (Jacobs et al.^25^). To check for potential foreign contamination of samples, “no template control” (DNA/RNA free water) and “no RT control” reactions were also included in the analysis (Supplementary Materials, Figs. S1, S2). To confirm our results, the amplicons were sequenced by the Molecular Biology Techniques Laboratory (Faculty of Biology, Adam Mickiewicz University) and compared with data available in a public database (NCBI). For each treatment, 3 biological replicates were used, and 3 technical repetitions were performed. The relative expression was calculated using the 2^−ΔΔCt^ method^26^.

### Lysozyme-like antimicrobial activity of haemolymph from *T. molitor*

The lysozyme-like antimicrobial activity of haemolymph from *T. molitor* was tested on the basis of the method described by Arce et al.^27^. The tested individuals were injected with 2 µL of physiological saline or a solution of physiological saline and Tenmo-TRP-7 at concentrations of 10^–7^ or 10^–5^ M. To activate the *T. molitor* immune system, 2 h after injection, the beetles were injected with 2 µL of a 10% physiological saline solution and attenuated *Staphylococcus aureus* (Sigma S2014, Saint Louis, Missouri, USA). Twenty-four hours after the physiological saline or neuropeptide injection, haemolymph samples (2 µL) were collected and transferred to 90 µL of ice-cold physiological saline and *Micrococcus luteus* solution (3 mg/10 mL; OD_600_ = 0.4, Sigma M3770-5G). Then, the samples were mixed and incubated at 37 °C for 30 min using a Thermomixer comfort 5355 (Eppendorf, Hamburg, Germany). After incubation, the samples were immediately chilled on ice, and the absorbance was checked (λ = 600 nm) using a BioSpectrometer kinetic (Eppendorf, Hamburg, Germany). The sample absorbance was compared to the absorbance of a physiological saline and *M. luteus* solution (blank; 0). The level of the absorbance reduction, i.e., the reduction in *M. luteus* content was used to indicate the lysozyme-like activity of the haemolymph. As a positive control, the antimicrobial activity of a physiological saline and lysozyme solution (Sigma L-7651, Saint Louis, Missouri, USA) was tested (0.1 mg/mL). At least 13 individuals were used in each of the treatments, and three independent replications were conducted.

### Statistical analysis

For the statistical analysis of physiological experiments, GraphPad Prism software was used (Adam Mickiewicz University licence, version 9.0.0 for Windows, GraphPad Software, San Diego, California USA, www.graphpad.com). Survival was analysed using the log-rank (Mantel–Cox) test. The outliers were defined using the ROUT method (Q = 1). The normality of the distribution was determined using the Shapiro–Wilk test. To check the homogeneity of variance, the Brown–Forsythe test and the Levene test were used. Normally distributed data were analysed with one-way ANOVA and a Student’s t test with Welch’s correction. Data with a non-normal distribution were analysed using the Mann–Whitney U test.

## Results and discussion

### Survival

**Figure 1:**
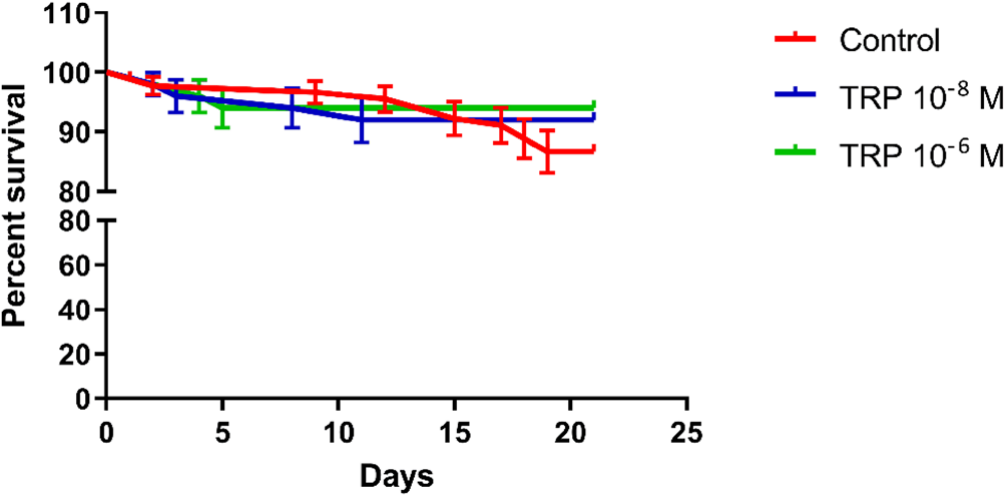
Survival curve of *T. molitor* males after the injection of physiological saline (control) and Tenmo-TRP-7 at concentrations of 10^–7^ and 10^–5^ M (final concentrations in insect bodies of 10^–8^ and 10^–6^ M, respectively). The values are given as the means ± SEM.

Over 21 days, we did not find any statistically significant differences between the control individuals and beetles treated with Tenmo-TRP-7 at concentrations of 10^–8^ and 10^–6^ M, which suggests that a single injection does not influence the lifespan of *T. molitor*. This result suggests a low cytotoxicity from the tested neuropeptide, even at the high concentration of 10^–6^ M (Fig. 1).

### General transcriptome information from *T. molitor*

We assembled the transcriptome from 20 libraries, each consisting of pooled samples from the fat body and haemocytes of *T. molitor*, after the injection of physiological saline or Tenmo-TRP-7 treatment at concentrations of 10^–8^ and 10^–6^ M. During the transcriptomic assay, 20.852.370–29.416.364 raw reads per library (average: 2.4814.596) were obtained. The average overall alignment rate was 80%. A total of 74–85% of reads uniquely mapped to the reference of the transcriptome assembly for *T. molitor*, as published by Johnston et al.^17^. The full transcriptome was submitted to the NCBI database (BioProject: PRJNA781435).

### General functional annotation of the transcriptome from *T. molitor*

**Figure 2:**
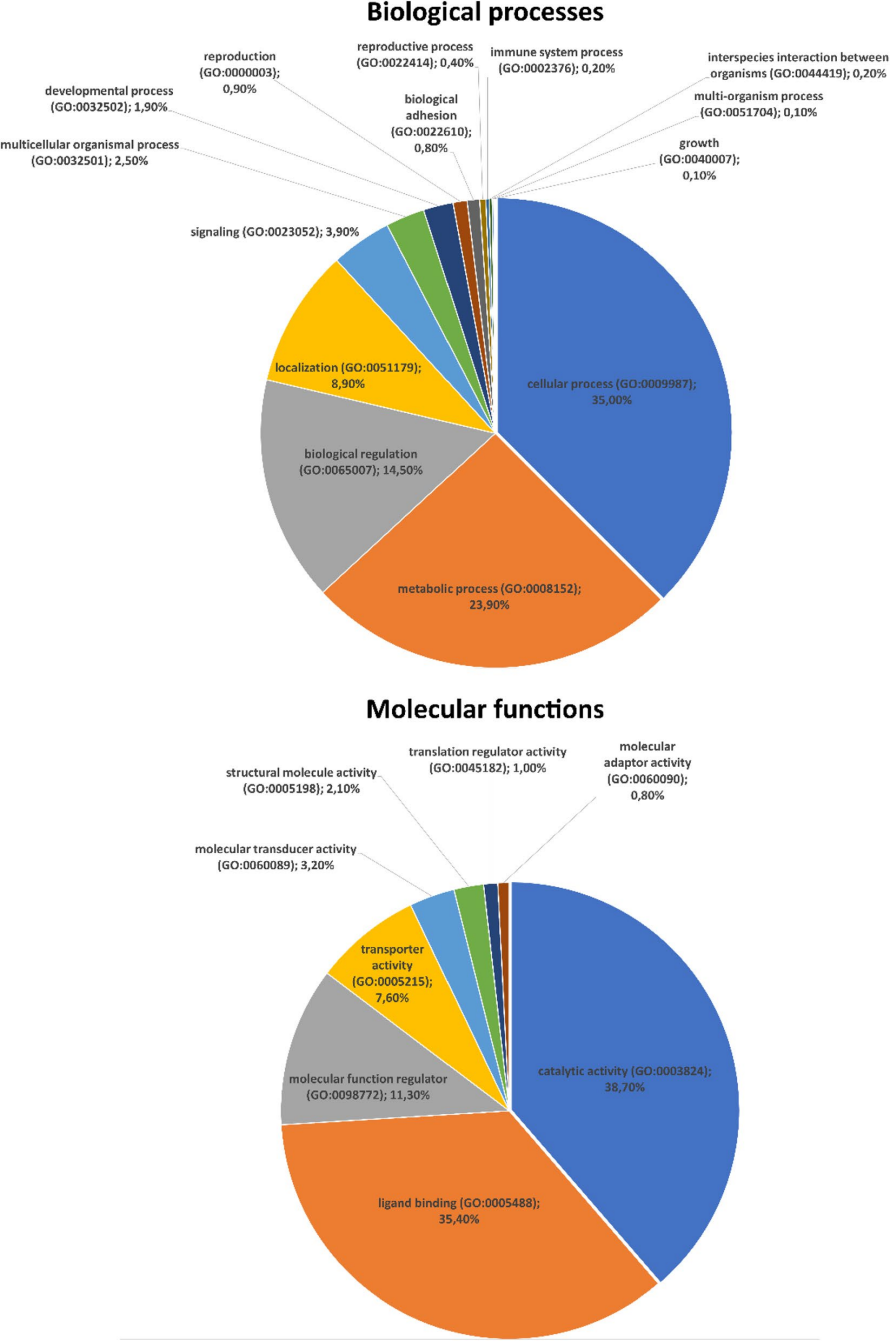
Gene ontology (GO) analysis based on the transcriptomic data of fat body and haemocytes from *Tenebrio molitor* was performed using the PANTHER database, and it included biological processes and molecular functions (https://www.pantherdb.org/).

The results of a gene ontology analysis on molecular functions and biological processes are shown in Fig. 2. In the case of biological functions, the most abundant GO terms were related to cellular processes (GO:0009987; 35%), metabolic processes (GO:0008152; 23.9%) and biological regulation (GO:0065007; 14.5%). The GO analysis associated with the molecular processes showed that the dominant GO terms were catalytic activity (GO:0003824; 38.7%), ligand binding (GO:0005488; 35.4%) and molecular function regulator (GO:0098772, 11.3%) (Fig. 2).

### Differences in the expression levels of genes 6 h after Tenmo-TRP-7 injection

#### GO enrichment analysis

**Figure 3:**
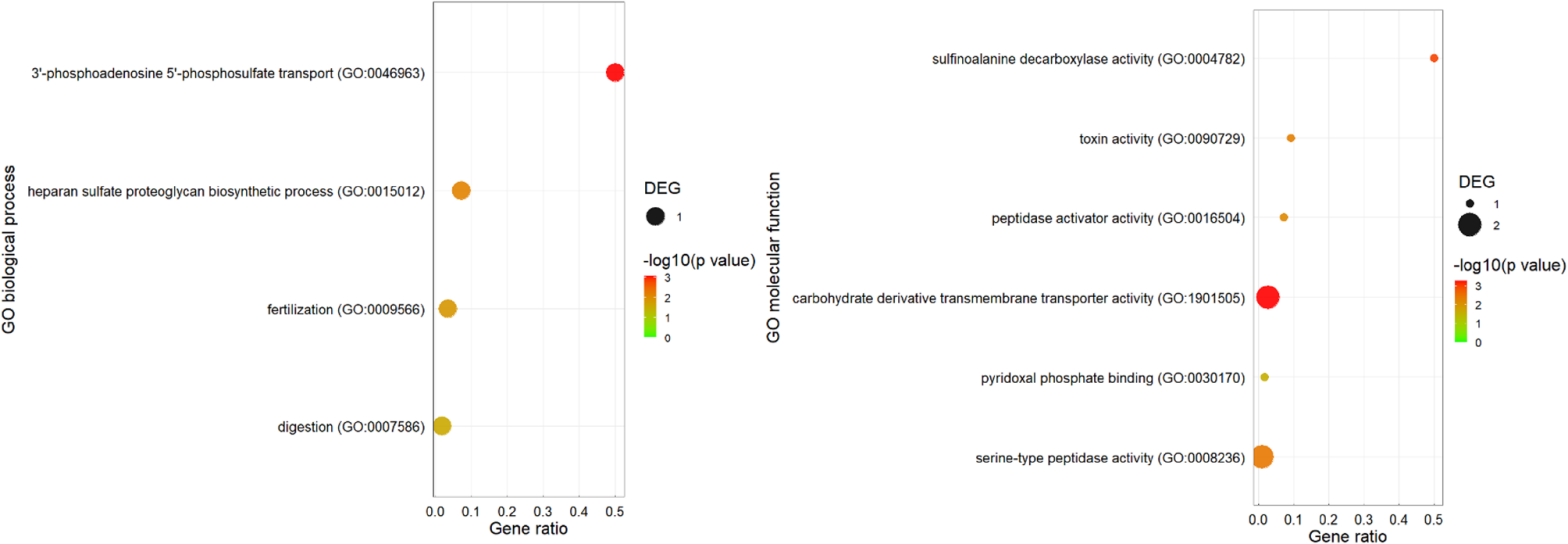
GO enrichment dot plot presenting changes in the expression level of different sets of genes observed 6 h after Tenmo-TRP-7 treatment at a concentration of 10^–8^ M. The identification of the most representative GO terms representing biological process and molecular function using REVIGO (http://revigo.irb.hr/) was performed (cut off < 0.05). The size of the dots represents the number of genes in the significantly differentially expressed genes (DEGs). Gene ratio’ is the percentage of total DEGs in the given GO term. For the graphical presentation of the obtained data, *ggplot2* (https://ggplot2.tidyverse.org) for RStudio was used (http://www.rstudio.com)^21,23,24^.

**Figure 4:**
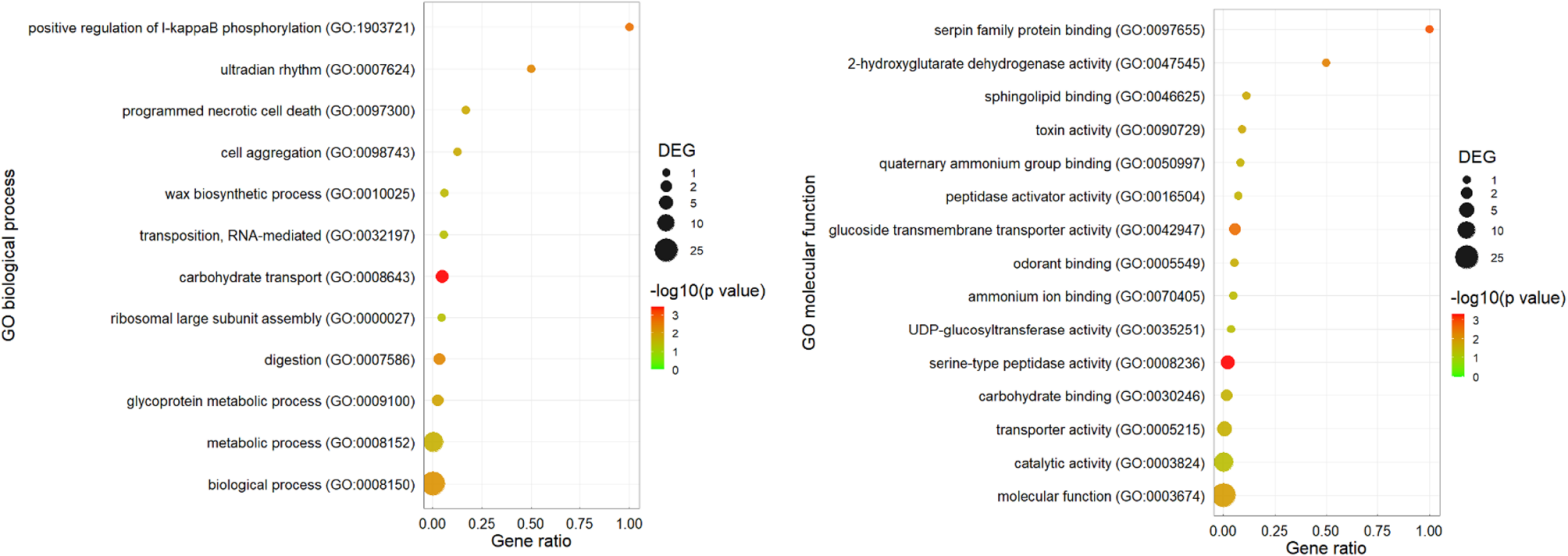
GO enrichment dot plot presenting changes in the expression level of different sets of genes observed 6 h after Tenmo-TRP-7 treatment at a concentration of 10^–6^ M. The identification of the most representative GO terms of biological process and molecular function using REVIGO (http://revigo.irb.hr/) was performed (cut off < 0.05). The size of the dots represents the number of genes in the significantly differentially expressed genes (DEGs). Gene ratio’ is the percentage of total DEGs in the given GO term. For the graphical presentation of the obtained data, *ggplot2* (https://ggplot2.tidyverse.org) for RStudio was used (http://www.rstudio.com)^21,23,24^.

The GO enrichment analysis showed that Tenmo-TRP-7 injection induced changes in the expression levels of various sets of genes after 6 h. Differences were observed for *biological processes, molecular functions,* and *cellular components* (Figs. 3, 4, Figs. S3, S4). GO term enrichment analysis indicated that Tenmo-TRP-7 participates in the regulation of metabolic processes. This regulation is associated with changes in the expression of genes classified to generally metabolic process (GO:0008152), digestion (GO:0007586) or carbohydrate transport (GO:1901505 and GO:0008643) (Figs. 3, 4). These results support previous research concerning the physiological role of TRPs in insects^1,28^. The results also suggest that Tenmo-TRP-7 may elicit effects directly and indirectly related to immune processes and cell death. These effects are observed after the application of both concentrations used here. In the case of the 10^–8^ M Tenmo-TRP-7 concentration, a differential expression of genes classified as serine-type peptidase activity terms (GO:0008236, molecular function) was noted, likely connected with the immune-regulatory role of Tenmo-TRP-7^29^ (Fig. 3). Six hours after Tenmo-TRP-7 treatment at a concentration of 10^–6^ M, GO enrichment analysis confirmed the participation of TRPs in the regulation of immune response and cell death, which was especially visible in the case of GO terms associated with biological processes and molecular functions. At this concentration, the most representative GO terms were positive regulation of Ikappa-B phosphorylation (GO:1903721), programmed necrotic cell death (GO:0097300) or serpins family protein binding (GO:0097655) (Fig. 4).

#### Differences in the expression levels of immune-related genes

The GO enrichment analysis was enriched by a detailed analysis of differentially expressed genes in the fat body and haemocytes 6 h after Tenmo-TRP-7 injection (Table 1). The presence of the neuropeptide at a concentration of 10^–8^ M led to a reduction in the expression levels of genes regulating the haemocyte activity. One of these genes is the gene encoding saccharopine dehydrogenase-like oxidoreductase, the overexpression of which is characteristic of the times before and after haemocyte spreading and encapsulation^30^. Moreover, the gene for a putative serine proteinase, one of the mediators of insect immune responses^29^, was also downregulated (Table 1).

**Table 1:**
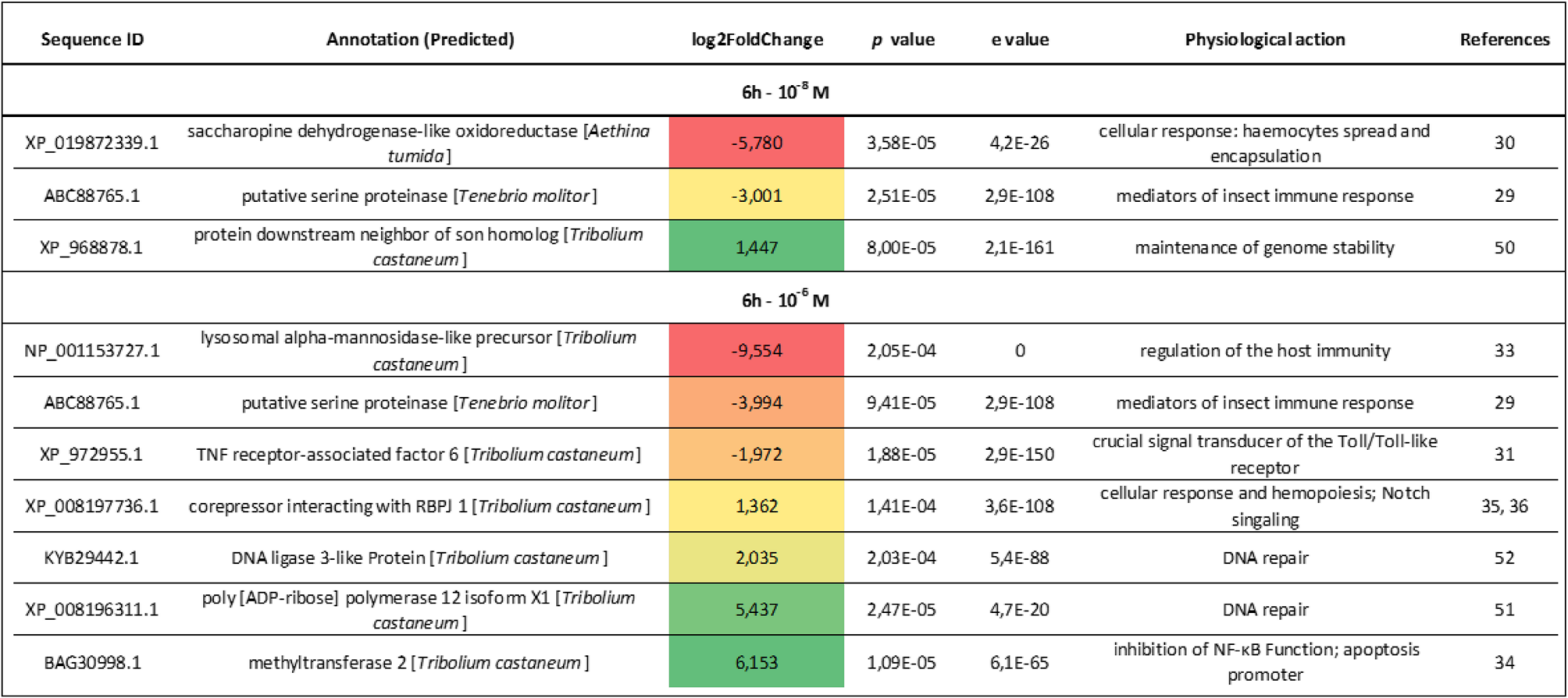
Differential expression of selected immune-related transcripts in the fat body and haemocytes of *T. molitor* 6 h after Tenmo-TRP-7 treatment at concentrations of 10^–8^ and 10^–6^ M.

Gradient from red to green: Fold change values represent the increased (green) or decreased (red) abundance of transcripts (adjusted *p* value of < 0.05) compared to the control individuals that were injected with physiological saline.

For the samples collected 6 h after injection, Tenmo-TRP-7 at a 10^–6^ M concentration also caused a significant downregulation of the putative serine proteinase gene. In addition, a reduction in the expression level of TNF receptor-associated factor 6, which is also involved in the regulation of the insect immune system as a signal transducer of the Toll/Toll-like receptor^31^, was found (Table 1). In beetles treated with 10^–6^ M Tenmo-TRP-7, the downregulation of the lysosomal alpha-mannosidase-like precursor gene was shown (Table 1). This precursor is involved in neutrophil degranulation in vertebrates^32^. In insects, lysosomal alpha-mannosidase, a component of *Bracon nigricans* venom, can be involved in the regulation of host immunity, especially for the recruitment of haemocytes for wound healing^33^. Additionally, the gene encoding methyltransferase 2, which inhibits NF-κB function, was strongly overexpressed^34^. Despite the changes that may indicate the inhibition of immune system functioning, the overexpression of a gene encoding a corepressor that interacts with the recombination signal binding protein for immunoglobulin kappa J 1 (RBPJ 1) was detected. This protein is a part of Notch signalling, which regulates insect development, but crosstalk with immune-related genes was also confirmed^35–37^.

These results are consistent with the results on haemocyte activity obtained by Urbański et al.^9^, which showed that the injection of Tenmo-TRP-7 led to decreasing numbers of haemocytes participating in phagocytosis. Additionally, a similar effect was observed in an in vitro experiment when neuropeptide was added to the physiological saline during haemocyte incubation on microscopic slides. Previously, we showed that 6 h after Tenmo-TRP-7 injection, the adhesion ability of haemocytes significantly decreased, which aligns with the differences in the expression of saccharopine dehydrogenase-like oxidoreductase^30,37^. The downregulation of a putative serine proteinase gene that mediates the immune response was also reported. The members of this superfamily participate in many immune processes, such as haemolymph clotting, melanotic encapsulation, antimicrobial peptide (AMP) induction, and cytokine activation^38^.

The differences between the two Tenmo-TRP-7 concentrations in the modulation of the expression levels of the different sets of immune-related genes observed here can be explained in a number of ways. In immunological studies, our knowledge is based only on data collected in research conducted on vertebrates after the application of SP, TKs homological to Tenmo-TRP-7. Many examples of dose-dependent SP actions on immune mechanisms in vertebrates have been reported^39^. For example, this neuropeptide might affect different regulatory cytokines^39^. Additionally, a similar dose-dependent SP activity was observed in the modulation of macrophage and mast cell functions^39–41^. These results are likely related to the fact that the effects of SP can be mediated by the C-terminal and N-terminal ends^39–41^. Recent studies on SP also connect dose-dependent actions of TKs with the regulation of the de/re-sensitization process. As suggested by Roosterman et al.^42^ and Vigna^43^, the phosphorylation of neurokinin 1 receptor (NK1R, receptor for SP) is strongly dependent on the concentration of SP. Research conducted on *Drosophila* TRPs seems to confirm this phenomenon in insects^44^. Research by Birse et al.^45^ and Poels et al.^46^ showed that TRPs can increase the intracellular calcium and cyclic AMP levels differently depending on the concentration. In addition, research conducted on, for example, the fly *Bactrocera dorsalis*, showed that the EC_50_ value for TRP oscillated at approximately 10^–8^ M, but the maximal response was observed at approximately 10^–5^ M^47^. We did not exclude the possibility that the effects observed at the highest concentration used here may also be related to the release of other neuropeptides in response to high concentrations of Tenmo-TRP-7 in the insect body. For example, Locmi-TRP-1, identified in *Locusta migratoria*, may modulate the release of AKH from the locust *corpora cardiaca.* This effect is known to be dose-dependent^48,49^.

#### Differences in the expression levels of genes involved in DNA repair and apoptosis

The GO enrichment analysis showed that Tenmo-TRP-7 injection not only influenced the expression levels of immune-related genes after 6 h but also changed the transcript levels of genes directly/indirectly related to DNA repair and apoptosis. After neuropeptide treatment at a concentration of 10^–8^ M, the overexpression of the protein downstream neighbour of son homolog gene was visible (Table 1). In humans, this protein is crucial to maintaining genome stability by protecting stalled or damaged replication forks^50^. At a concentration of 10^–6^ M, more changes related to the genes participating in DNA repair were reported. Under these conditions, DNA ligase 3-like protein and poly [ADP-ribose] polymerase were overexpressed, and they are involved in the activation and modulation of DNA repair machinery^51,52^ (Table 1). The results related to DNA repair are consistent with the results presented by Urbański et al.^9^: administering Tenmo-TRP-7 led to a significant decrease in DNA damage in *Tenebrio* haemocytes after 6 h, but only at a concentration of 10^–8^ M. These results also indicate functional homology in TK signalling in vertebrates and insects because SP can delay neutrophil and macrophage apoptosis^53,54^. In contrast, we previously reported that 6 h post-injection, Tenmo-TRP-7 at a 10^–6^ M concentration decreased the level of DNA integrity in *Tenebrio* haemocytes compared to control individuals. This finding may be a result of the overexpression of the previously mentioned methyltransferase 2, which is also a promoter of apoptosis^34^ (Table 1).

#### RT-qPCR analysis

**Figure 5:**
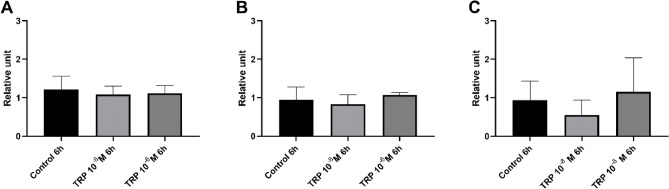
Reverse transcription quantitative PCR (RT-qPCR) analysis of the expression level of genes encoding selected antimicrobial peptides ((**A**) attacin 2; (**B**) tenecin 3) and the Toll receptor (**C**) in immune-related tissues (pooled fat body and haemocytes) 6 h after Tenmo-TRP-7 application at concentrations of 10^–8^ and 10^–6^ M. The values are the means ± SEM.

The expression levels of genes encoding attacin 2, tenecin 3 and the Toll receptor did not change significantly 6 h after the application of Tenmo-TRP-7 (Fig. 5). The results of the RT-qPCR assay are consistent with the transcriptomic data, which did not show changes in the expression levels of these selected immune-related genes.

### Differences in the expression of genes 24 h after Tenmo-TRP-7 injection

#### GO enrichment analysis

**Figure 6:**
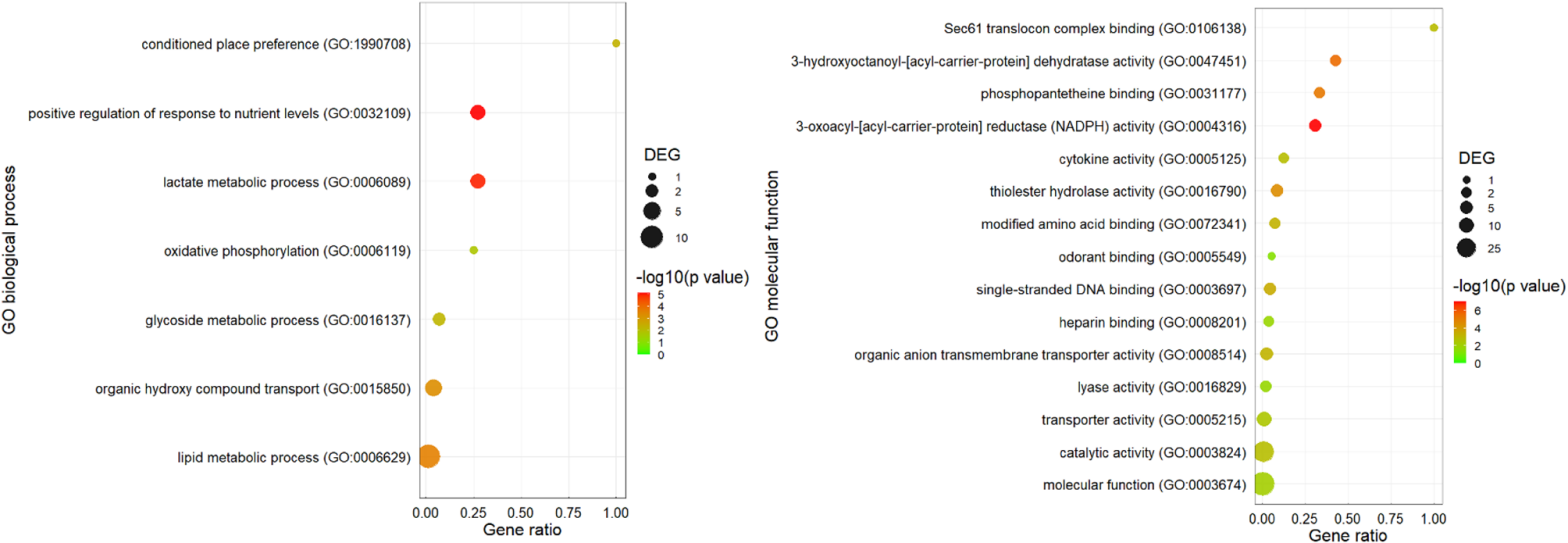
GO enrichment dot plot presenting a comparison of transcriptomic data between control individuals injected with physiological saline and 24 h after Tenmo-TRP-7 treatment at a concentration of 10^–8^ M. The identification of the most representative GO terms of biological process and molecular function using REVIGO (http://revigo.irb.hr/) was performed (cut off < 0.05). The size of the dots represents the number of genes in the significantly differentially expressed genes (DEGs). Gene ratio' is the percentage of total DEGs in the given GO term. For the graphical presentation of the obtained data, *ggplot2* (https://ggplot2.tidyverse.org) for RStudio was used (http://www.rstudio.com)^21,23,24^.

**Figure 7:**
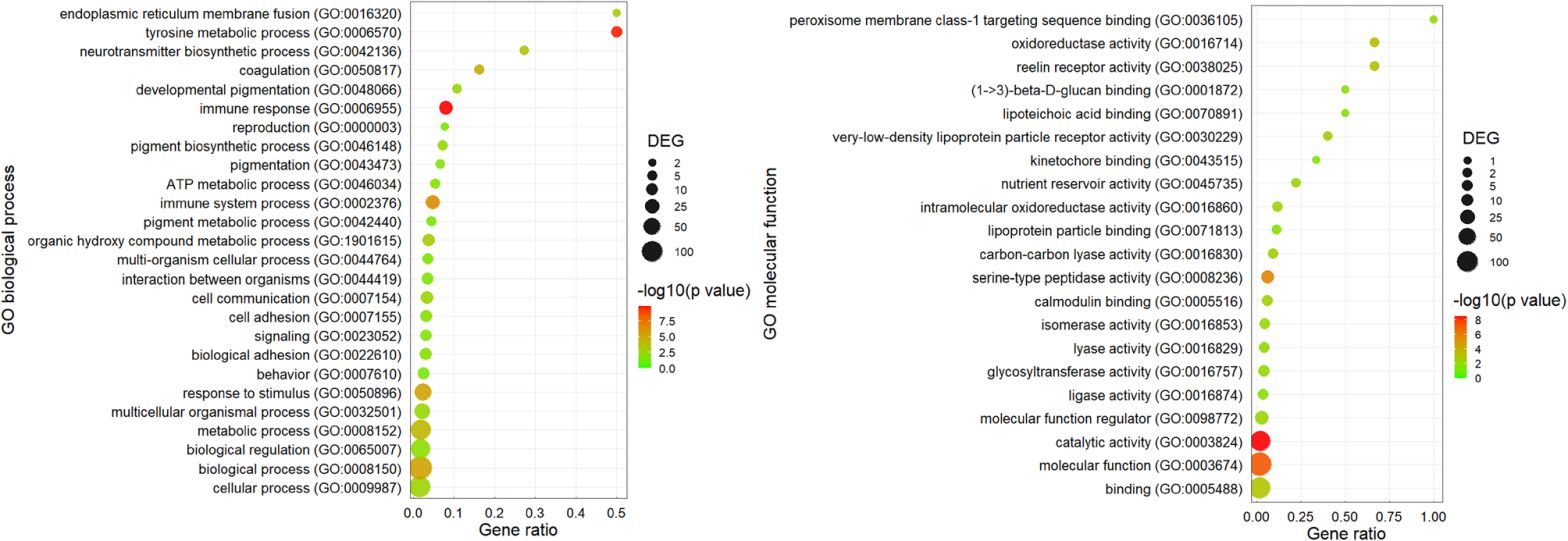
GO enrichment dot plot presenting changes in the expression levels of different sets of genes observed 24 h after Tenmo-TRP-7 treatment at a concentration of 10^–6^ M compared to the control. The identification of the most representative GO terms for biological process and molecular function using REVIGO (http://revigo.irb.hr/) was performed (cut off < 0.05). The size of the dots represents the number of genes in the significantly differentially expressed genes (DEGs). Gene ratio’ is the percentage of total DEGs in the given GO term. For the graphical presentation of the obtained data, *ggplot2* (https://ggplot2.tidyverse.org) for RStudio was used (http://www.rstudio.com)^21,23,24^.

Similar to the differences observed 6 h after the application of Tenmo-TRP-7, the GO enrichment analysis clearly showed that after 24 h, the tested neuropeptide elicited numerous changes in the expression levels of different sets of genes closely related to metabolic processes (Figs. 6, 7). Twenty-four hours after the application of Tenmo-TRP-7, other processes started to become more pronounced, such as biological processes and molecular functions related to energy metabolism (for example, at a concentration of 10^–8^ M: oxidative phosphorylation, GO:0006119; at a concentration of 10^–6^ M, ATP metabolic process, GO:0046034), and response to different stimuli, including stress responses (for example, differences in the expression of genes associated with catalytic activity (GO:0003824) at both tested concentrations or the tyrosine metabolic process (GO:0006570) at a concentration of 10^–6^ M)^55,56^.

Despite these changes, GO enrichment analysis clearly showed that 24 h after the application of Tenmo-TRP-7, the immunomodulatory impact increased (Figs. 6, 7). At a concentration of 10^–8^ M, one of the most representative GO terms was cytokine activity (GO:0005125, molecular function) (Fig. 6). In the 10^–6^ M Tenmo-TRP-7 treatment, the increasing number of GO terms related to immune system functioning was clear (Fig. 7). Under this treatment, the enhanced significance of immune processes was connected to the increasing number of differentially expressed genes classified, for example, as immune response (GO:0006955, biological process), immune system process (GO:0002376, biological process), coagulation (GO:0050817, biological process), pigmen biosynthesis process (GO:0046148), pigment metabolic process (GO:0042440, biological process), cell adhesion (0007155, biological process) or serine-type peptidase activity (GO:0008236, molecular function) (Fig. 7).

#### Differences in the expression levels of immune-related genes

The detailed bioinformatic analysis of transcriptomic data from immune-related tissues 24 h after Tenmo-TRP-7 injection showed statistically significant changes in the expression of a wide spectrum of immune-related genes (Table 2).

**Table 2:**
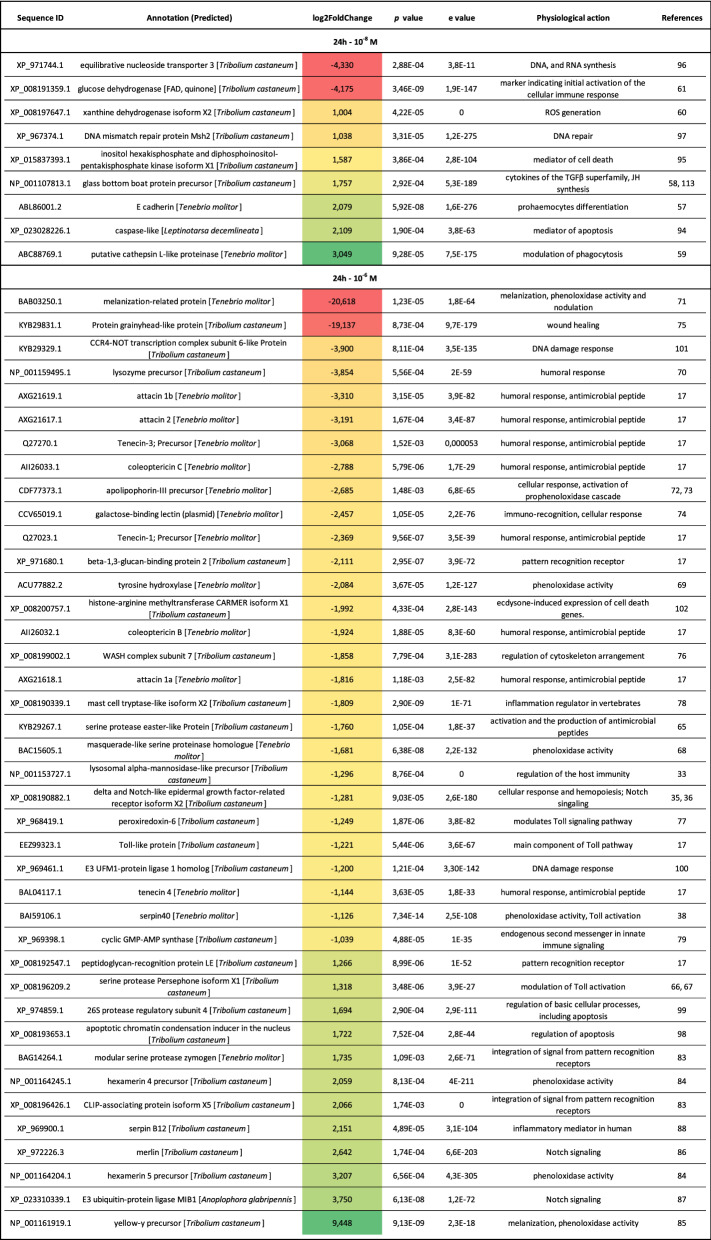
Differential expression of selected immune-related transcripts in the fat body and haemocytes of *T. molitor* 24 h after Tenmo-TRP-7 treatment at concentrations of 10^–8^ and 10^–6^ M.

Gradient from red to green: Fold change values represent the increased (green) or decreased (red) abundance of transcripts (adjusted *p* value of < 0.05) compared to the control individuals injected with physiological saline.

A detailed transcriptomic analysis showed that 10^–8^ M Tenmo-TRP-7 significantly changed the expression level of genes mostly involved in the regulation of the cellular response (Table 2). Under this treatment, compared to the control individuals, we observed an overexpression of genes encoding cathepsin L-like proteinase (the degradation of internalized material in phagocytic cells), E cadherin (limitation of pro-haemocyte differentiation) and glass bottom boat protein precursor (a cytokine of the TGFβ superfamily)^57–59^ (Table 2). A slight but significant increase in the expression levels of a gene that participates in the generation of damage-causing reactive oxygen species (ROS), xanthine dehydrogenase, was observed^60^. Additionally, the gene encoding glucose dehydrogenase [FAD, quinone] was downregulated, and it is a marker of the initial activation of the cellular immune response^61^ (Table 2). The regulation of the previously mentioned different set of immune-related genes confirms our previous findings concerning dose- and time-dependent effects elicited by TRPs but also by other neuropeptides, such as AKHs^9,62–64^ (Table 2).

Tenmo-TRP-7 treatment at a concentration of 10^–6^ M led to significant changes in the expression levels of many genes related to immune system functioning, including genes encoding AMPs and genes related to the activity of PO system (Table 2). Compared to the control individuals, Tenmo-TRP-7 at a 10^–6^ M concentration caused a downregulation of genes encoding the AMPs attacins (1a, 1b and 2), tenecins (precursors for tenecins 1 and 3, tenecin 4) and coleoptericins (C and D) (Table 2). Not only did the expression levels of AMP genes decrease, but other genes encoding proteins connected with AMP synthesis, such as beta-1,3-glucan-binding protein 2, Toll-like protein, and serine protease easter-like protein^17,65^, were also repressed. Interestingly, the gene for the serine protease Persephone (which participate in Toll activation) was overexpressed^66^. Also, research conducted by Issa et al.^67^ showed that Persephone belongs to a danger pathway activated by elevated proteolytic activities that can lead to the activation of Toll signalling. Additionally, other important components of the humoral, but also the cellular response were inhibited. For example, the expression levels of genes encoding lysozyme precursors and genes involved in the melanization process, including PO system activity (melanization-related protein, tyrosine hydroxylase, masquerade-like serine proteinase homologue, serpin 40)^38,68–71^, were reduced. The expression level of the apolipophorin-III precursor gene, which is involved in the regulation of cellular responses and PO system activity, was significantly decreased^72,73^. The transcriptomic analysis also showed a decrease in the expression of other genes involved in the regulation of immune system activity, such as genes for grainyhead-like protein (regulation of wound healing), WASH complex subunit 7 (regulation of cytoskeleton arrangement during cell migration), delta and Notch-like epidermal growth factor-related receptor and galactose-binding lectin (involved in immuno-recognition) or lysosomal alpha-mannosidase-like precursor (haemocyte recruitment)^33,35,74–76^ (Table 2). There are other genes that contribute to immune system regulation, but their immunomodulatory role has not been confirmed in insects, and they were also downregulated. For example, the mast cell tryptase-like gene, which is involved in vertebrates, is involved in regulating inflammation, peroxiredoxin 6, which modulates Toll signalling in red swamp crayfish, and cyclic GMP-AMP synthase, the endogenous second messenger in innate immune signalling by cytosolic DNA, were all downregulated^32,77–79^ (Table 2).

All these changes in the expression level of genes associated with the *T. molitor* immune response indicate a strong inhibition of immune system activity by TRPs during extended stress conditions, consistent with the overexcitation hypothesis^62,80,81^. This hypothesis assumes that the high concentration of hormones, characteristic of prolonged stress conditions, should result in a reduction in the activity of the immune system. This mechanism has been suggested to be crucial for the protection of host tissues against autoimmunological injuries^62,80–82^. It should also be noted that some of the genes involved in the regulation of insect immune system activity were upregulated. Compared to the control group, the slight overexpression of the peptidoglycan-recognition protein LE gene was observed (Table 2). Genes involved in the integration of signals from pattern recognition receptors (genes encoding modular serine protease zymogen and CLIP-associating protein)^83^ were overexpressed. Interestingly, the upregulation of some genes related to the melanization process and PO system activity was also found (Table 2). Twenty-four hours after the application of 10^–6^ M Tenmo-TRP-7, the expression levels of yellow-y precursor and hexamerin precursors (4 and 5) significantly increased^84,85^. Additionally, overexpression of the Notch pathway (E3 ubiquitin-protein ligase, merlin) and serpin B12 (which plays an important role as an inflammatory regulator in humans) was observed^86–88^. The potential simultaneous inhibition and stimulation of some of the immune system components support previous research concerning the possible role of TRPs in the modulation of the *Tenebrio* immune system as well as other research on the influence of hormones on insect physiology^9^. According to the hypothesis proposed by Adamo^62^, this situation may be explained by the adaptive reconfiguration of the immune system. It is manifested by switching the function of some elements participating in immune process regulation, which causes the inhibition of specific immune mechanisms to be compensated by the stimulation of other parts of this system^62^.

The differences in transcription 24 h after applying Tenmo-TRP-7 at different concentrations may be explained in a similar way as the presence of the dose-dependent changes observed 6 h after neuropeptide application. The dose-dependent modulation of the expression level of immune-related genes is almost certainly associated with the different activation of the TRP receptor and/or the influence of other neuropeptides, which can be released in response to the presence of TRPs. Moreover, the time-dependent action of Tenmo-TRP-7 can be explained by a general mode of action of neuropeptides. Based on the research by Diniz et al.^89^ conducted on the TRPs identified in *Triatoma infestans,* the time to the full degradation of TRPs oscillated at approximately 120 min. The results in vertebrate TKs, especially SP, are comparable to those obtained in research on insects^90^. However, neuropeptides usually bind to GPCRs (G protein-coupled receptors) and elicit second messenger cascades to modulate cell activity on longer timescales^91,92^. Research conducted on vertebrate SP found time-dependent activity^39^. For example, research by Scicchitano et al.^93^ showed that the time of incubation is crucial in determining the effects of SP on human lymphocytic responses. The inhibitory response was observed after 24 h of incubation with SP, but no effect was found after 48 h of treatment^93^.

#### Differences in the expression levels of genes involved in DNA repair and apoptosis

We also found significant changes in the expression of genes involved in DNA repair and apoptosis 24 h post-treatment (Table 2). In the 10^–8^ M Tenmo-TRP-7 treatment, primarily differences related to apoptosis were observed (Table 2). This observation is linked to the overexpression of caspase-like protein in addition to inositol hexakisphosphate and diphosphoinositol-pentakisphosphate kinase, which are the physiological mediators of cell death^94,95^. The downregulation of the gene encoding equilibrative nucleoside transporter 3, which is crucial for DNA and RNA synthesis, was also noted^96^. However, slight overexpression of the DNA mismatch repair protein Msh2 was found^97^ (Table 2). In the 10^–6^ M Tenmo-TRP-7 treatment, we observed the overexpression of 26S protease regulatory subunit 4 and an apoptotic chromatin condensation inducer in the nucleus, which are likely involved in regulating apoptosis^98,99^. The genes for CCR4-NOT transcription complex subunit 6-like protein and E3 UFM1-protein ligase 1 homolog, which is related to the DNA damage response^100,101^, were downregulated. The gene for histone-arginine methyltransferase CARMER was also downregulated. This methyltransferase is important for the modulation of the ecdysone-induced expression of cell death genes^102^. In addition, peroxiredoxin 6, which protects DNA against damage associated with oxidative stress^77^, was downregulated (Table 2). In accordance with our previously published results^9^, 24 h after Tenmo-TRP-7 injection, changes were observed in the expression levels of genes involved in regulating apoptosis. Our previous research clearly demonstrated that 24 h after testing neuropeptide application, compared to the control, a higher level of DNA damage in *Tenebrio* haemocytes was observed^9^. This finding was especially visible in the case of Tenmo-TRP-7 treatment at a concentration of 10^–8^ M, in which, as current research showed, the overexpression of the gene encoding caspase-like protein was reported^9^.

#### RT-qPCR analysis

**Figure 8:**
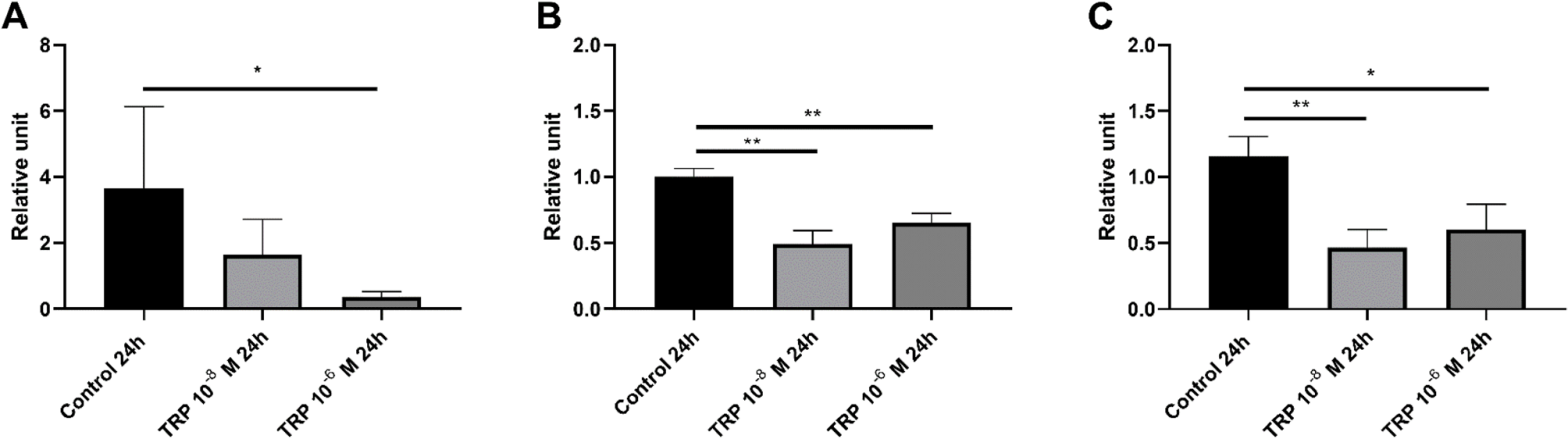
Reverse transcription quantitative PCR (RT-qPCR) analysis on the expression levels of genes encoding selected antimicrobial peptides ((**A**) attacin 2; (**B**) tenecin 3) and the Toll receptor (**C**) in immune-related tissues (pooled fat body and haemocytes) 24 h after applying Tenmo-TRP-7 at concentrations of 10^–8^ and 10^–6^ M. Values are the means 土 SEM. *p ≤ 0.05; **p ≤ 0.01.

The RT-qPCR analysis supports the transcriptomic data. Applying Tenmo-TRP-7 led to significant changes in the expression levels of the genes encoding attacin 2, tenecin 3 and Toll receptor (Fig. 8). Similar to the transcriptomic data, the neuropeptide caused a decrease in the expression levels of selected immune genes 24 h after its application at a concentration of 10^–6^ M (Mann Whitney U test; attacin 2, U = 4.00; *p* ≤ 0.05; t test with Welch’s correction; tenecin 3, t = 3.70; *p* ≤ 0.01; and Toll, t = 2.24; *p* ≤ 0.05). However, significant downregulation of the tenecin 3 and Toll receptor genes was also observed after Tenmo-TRP-7 treatment at a concentration of 10^–8^ M (t test with Welch’s correction; tenecin 3, t = 4.19; *p* ≤ 0.01; and Toll, t = 3.28; *p* ≤ 0.01) (Fig. 8B,C). The observed variances between the RT-qPCR assay and transcriptomic data may be related to the different accuracies of these two methods^103^.

### Differences in the expression levels of genes that may indirectly influence *T. molitor* immune system activity

The comparative transcriptomic analysis of *Tenebrio* immune-related tissue revealed genes that are directly involved in regulating immune system functioning. We now report the differential expression of genes that are likely to indirectly influence the immune system. The candidates are genes involved in regulating stress responses (including detoxification and nociception), metabolism, circadian clock, and hormone biosynthesis. All this information is summarized in Table 3.

**Table 3:**
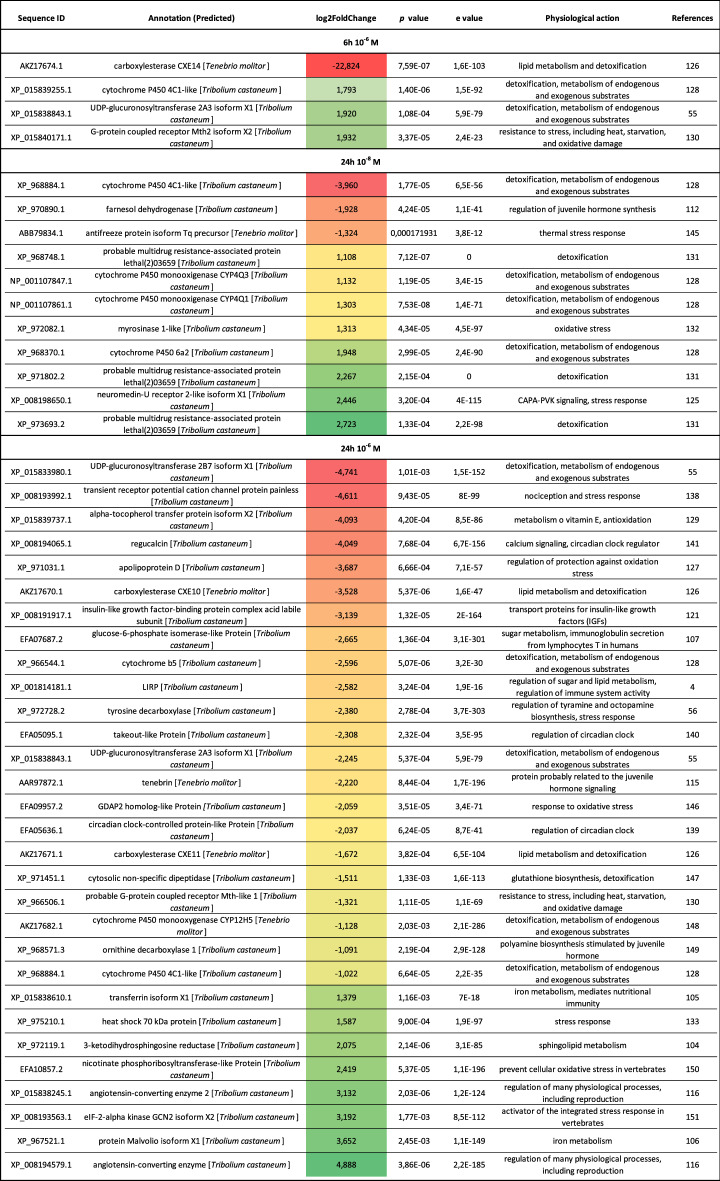
Statistically significant differences in the expression levels of selected metabolic- and stress-related genes in the immune-related tissue of *T. molitor* collected 6 and 24 h after Tenmo-TRP-7 treatment at concentrations of 10^–8^ and 10^–6^ M.

Gradient from red to green: Fold change values represent the increased (green) or decreased (red) abundance of transcripts (adjusted *p* value of < 0.05) compared to the control individuals injected with physiological saline.

The changes in the expression level of metabolism-related genes were visible in genes involved in regulating lipid and sugar metabolism (Figs. 3, 4, 6, 7). Some changes were also visible in the expression of genes participating in energy metabolism, protein synthesis and degradation (Figs. 3, 4, 6, 7). The current literature shows that some of these genes can be indirectly involved in modulating immune system functioning^104–106^. A gene encoding glucose-6-phosphate isomerase was downregulated 24 h after applying Tenmo-TRP-7 at a concentration of 10^–6^ M (Table 3). This protein participates in glycolysis and glyconeogenesis but also immunoglobulin secretion from T-lymphocytes in humans^107^. Under this concentration, genes for 3-ketodihydrosphingosine reductase (sphingolipid metabolism may be important for immune functioning), transferrin and Malvolio protein (crucial for the regulation of iron homeostasis and immune system activity)^104–106^ were also significantly upregulated (Table 3).

Hormone biosynthesis was also affected by our treatments. Across all treatments, we observed differences in the expression levels of genes associated with juvenile hormones (JHs) biosynthesis and/or JH signalling. In addition to their regulatory role in development and reproduction, JHs may also act as immune suppressors^108–110^. However, some of the published data may also suggest an immunostimulatory role for JH^111^. Tenmo-TRP-7 at a concentration of 10^–8^ M led to the upregulation of genes participating in JH biosynthesis (farnesol dehydrogenase and the previously mentioned glass bottom boat protein precursor)^58,112,113^ (Tables 2, 3). Twenty-four hours after Tenmo-TRP-7 treatment at a concentration of 10^–6^ M, a decreasing level of expression of the Tenebrin gene (a protein likely involved in JHs and ecdysone signalling) and ornithine decarboxylase (its activity is stimulated by JH) were observed^114,115^ (Table 3).

Angiotensin converting enzyme (ACE), which is closely associated with the regulation of reproduction, development and hormone biosynthesis^116^, was overexpressed. Interestingly, research conducted by Macours et al.^117^ showed that ACE can be important for haemocyte activity because bacterial infection led to the increased expression of a gene for ACE in the haemocytes of desert locust. Wang et al.^116^ supported these results experimentally and showed elevated transcript levels of the ACE gene in the fat body during viral infection. Additionally, the application of 10^–6^ M Tenmo-TRP-7 after 24 h caused an increase in the expression levels of genes encoding ACE and ACE2 (Table 3). However, this finding may be related to the fact that ACE is required for TRP degradation in insects^118^.

An interesting finding is that in tissues collected 24 h after neuropeptide application at a concentration of 10^–6^ M, the gene encoding insulin-related peptides (LIPR-lGF_insulin_bombyxin_like domain-containing protein) was significantly downregulated (Table 3). This result supports the hypothesis about a close connection between TRPs and ILP signalling^4,119^. The inhibition of ILP signalling can cause multiple changes in insect physiology ranging from sugar and lipid metabolism to the direct and indirect modulation of immune system functioning^2,3,5^. We also found the downregulation of the gene encoding the insulin-like growth factor-binding protein complex acid labile subunit (Table 3). Insulin-like growth factor-binding proteins are a group of secreted proteins that serve as transport proteins for insulin-like growth factors (IGFs) that also influence the immune system^120,121^. Notably, the gene for tyrosine decarboxylase (TDC) was also downregulated (Table 3). Tyrosine decarboxylase, an enzyme catalysing the first decarboxylation step in the biosynthesis of tyramine and octopamine, is extremely important in the modulation of insect homeostasis during the stress response, including the modulation of insect metabolism and immune system functioning^56,122^. The decreased expression level of the TDC gene may lead to the inhibition of the immune response after Tenmo-TRP-7 administration because octopamine can enhance phagocytosis and AMP synthesis^123,124^. The importance of this observation is highlighted by the fact that one of the most representative GO terms was tyrosine metabolism process (Fig. 7). Interestingly, 24 h post-injection, 10^–8^ M Tenmo-TRP-7 modulated other hormonal signalling related to stress because the overexpression of the neuromedin U/CAPA-PVK receptor gene was observed (Table 3). CAPA-PVK signalling is primarily involved in the regulation of ion homeostasis, but our current research suggests that this group of neuropeptides could be involved in regulating the cellular response and haemocyte adhesion ability^125^.

Under all Tenmo-TRP-7 treatments, differences in the expression of other genes involved in the stress response were reported (Table 3). In particular, differences in the expression levels of genes participating in the detoxification and/or metabolism of endogenous substances were observed. This participation included genes for cytochrome P450 or the multidrug resistance-associated protein lethal and genes related to oxidative stress responses (for example, genes for G-protein coupled receptor Mth-like 1, alpha-tocopherol transfer protein or apolipoprotein D)^55,126–132^ (Table 3). Moreover, 10^–6^ M Tenmo-TRP-7 caused the overexpression of heat shock 70 kDa protein (Hsp70) 24 h after its administration. Research conducted by Tang et al.^133^, for example, confirmed that Hsp70 also plays an essential role in regulating insect immune system activity.

Nociception is closely associated with the functioning of transient receptor potential channels (TRP channels). Tachykinins are a key component of nociception via the modulation of TRP channel activity^134^. Current research clearly shows that the activity of these channels is also required to modulate pathogen recognition and inflammation^135,136^. TRP channels are evolutionarily conserved structures that are involved in nociception and the modulation of different physiological processes in insects^137^. We found a decrease in the expression level of the gene encoding the TRP channel protein painless^138^ 24 h after Tenmo-TRP-7 treatment at a concentration of 10^–6^ M (Table 3). TRP channels are also strongly involved in regulating the circadian cycle, consistent with the finding that other genes involved in the modulation of this process were downregulated 24 h after the application of Tenmo-TRP-7 (see also Wolfgang et al.^137^). Our comparative transcriptomic analysis showed that the tested neuropeptide injection at the highest concentration led to a decrease in the expression level of genes encoding regucalcin, takeout-like protein, and circadian clock-controlled protein-like protein^139–141^. Recent studies have shown that genes related to the control of circadian clock genes are required to modulate immune system activity, including cellular and humoral responses^142,143^. In addition, research conducted on the Pacific oyster *Crassostrea gigas* showed that regucalcin can suppress the apoptosis of haemocytes by regulating caspase-3 activity and nitric oxide (NO) production^144^.

### Lysozyme-like antimicrobial activity of *T. molitor* haemolymph

**Figure 9:**
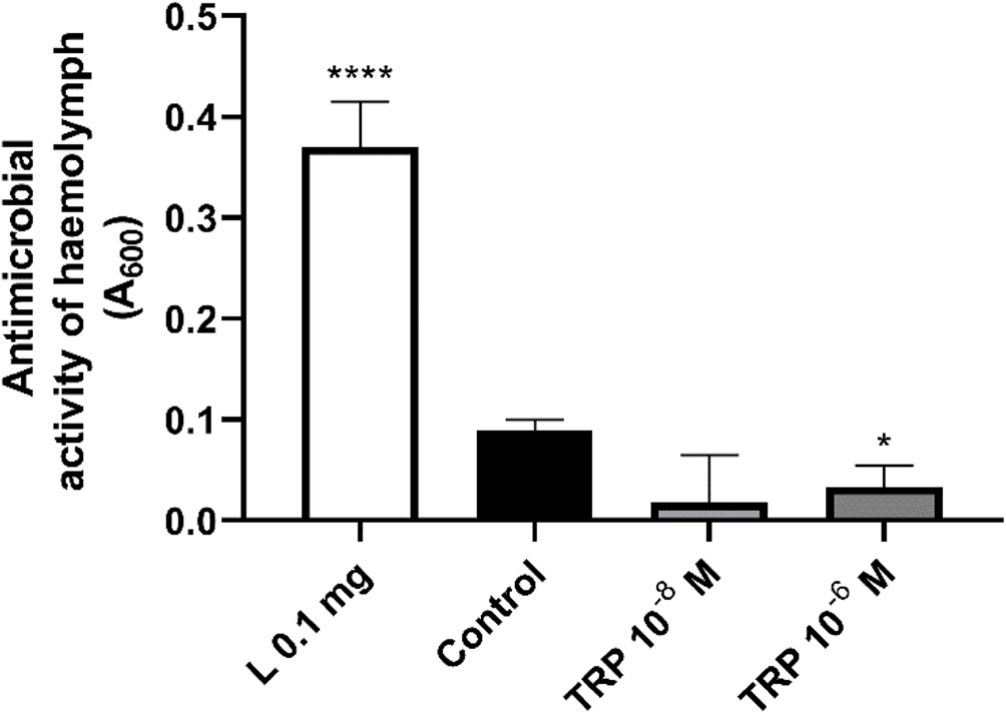
Lysozyme-like antimicrobial activity of *T. molitor* haemolymph against *Micrococcus luteus* when collected from control individuals injected with physiological saline (Control) or a solution of physiological saline and Tenmo-TRP-7 at concentrations of 10^–7^ and 10^–5^ M (for final concentrations of 10^–8^ and 10^–6^ M, respectively). L 0.1 mg, positive control, solution of physiological saline and lysozyme at a concentration of 0.1 mg/mL. To activate the immune system, beetles were injected with attenuated *Staphylococcus aureus* 2 h after Tenmo-TRP-7 administration. For better visualization of the observed differences, the obtained values were reversed. Asterisks indicate statistically significant differences compared to the control individuals; *p ≤ 0.05; ****p ≤ 0.0001; values are given as the means ± SEM.

To confirm that the reported changes in the expression level of immune-related genes have a significant impact on the activity of *T. molitor* immune mechanisms, the lysozyme-like antimicrobial activity of the haemolymph was analysed. The results showed statistically significant differences in the antimicrobial activity of *T. molitor* haemolymph after Tenmo-TRP-7 injection (one-way ANOVA, df = 3, 49; F = 16.03; *p* ≤ 0.0001) (Fig. 9): neuropeptide application led to a decrease in the lytic activity of *T. molitor* haemolymph against *M. luteus*. Despite the differences between the positive control (lysozyme 0.1 mg/mL), the inhibition of lysozyme-like antimicrobial activity in haemolymph was observed only in the comparison of the control group to the individuals treated with Tenmo-TRP-7 at a concentration of 10^–6^ M (t test with Welch’s correction, t = 2.30; *p* ≤ 0.05). These results are consistent with the previously mentioned overexcitation hypothesis and our previously published data^9^. Moreover, the antimicrobial assay also supports the presented transcriptomic analysis, which suggests that the observed changes in immune system functioning might be a result of the downregulation of immune-related genes, especially genes for lysozyme precursor and antimicrobial peptides as well as changes associated with genes participating in the regulation of metabolism and stress response (Tables 1, 2, 3). The lack of a significant inhibition of lysozyme-like activity after injecting 10^–8^ M Tenmo-TRP-7 does not indicate a lack of immunomodulatory properties for TRP at lower concentrations. Our previous research clearly showed that 10^–8^ M Tenmo-TRP-7 can modulate the haemocyte adhesion ability, which can also affect the activity of the *T. molitor* immune system^9^. Moreover, the transcriptomic data also confirmed that 10^–8^ M Tenmo-TRP-7 mostly modulated the cellular response.

## Conclusions

Our results shed new light on the regulation of the insect immune system by neuropeptides such as Tenmo-TRP-7 and the functional homology of TK signalling across different animal phyla. The comparative transcriptomic analysis confirmed previously published results and hypotheses on the time- and dose-dependent action of TRPs on insect immune system activity. The immunomodulatory effect was also observed in the analysis of lysozyme-like antimicrobial properties of *Tenebrio* haemolymph after the injection of Tenmo-TRP-7.

Knowledge about the hormonal regulation of basic physiological processes and factors that lead to immune deficiency in *T. molitor*, one of the storage pests, may be useful for developing new, specific and biosafe methods of pest control. In addition, due to confirmed structural and functional homology between TKs and TRPs, the presented results may be helpful for searching new alternative models in biomedical research for the study of hormonal regulation in innate immune function.

## Supplementary Information

Supplementary Information.

## Data Availability

The transcriptomic data were submitted to the NCBI database (BioProject: PRJNA781435; https://www.ncbi.nlm.nih.gov/bioproject/PRJNA781435). The rest of the datasets used during the current study are available from the corresponding author on reasonable request.
