# Supplementary material for: Tachykinin-related peptides modulate immune-gene expression in the mealworm beetle Tenebrio molitor L
Source: Sci Rep. 2022 Oct 14;12:17277. doi: 10.1038/s41598-022-21605-6 (PMC9568666; doi:10.1038/s41598-022-21605-6)
Supplement: Supplementary file 1 — Supplementary Information. [file 41598_2022_21605_MOESM1_ESM.docx]

**Supplementary materials: Tachykinin-related peptides modulate immune-gene expression in the mealworm beetle *Tenebrio molitor* L.**

Urbański A.^1*^, Johnston P.^2,3^, Bittermann E.^4^, Keshavarz M.^4^, Paris V.^4,5^, Walkowiak-Nowicka K.^1^, Konopińska N.^1^, Marciniak P.^1^, Rolff J.^4, 6^

^1^Department of Animal Physiology and Developmental Biology, Adam Mickiewicz University, Poznań, Poland

^2^Berlin Centre for Genomics in Biodiversity Research, Berlin, Germany,

^3^Leibniz-Institute of Freshwater Ecology and Inland Fisheries (IGB), Berlin, Germany

^4^Evolutionary Biology, Institute for Biology, Freie Universität Berlin, Berlin, Germany

^5^Bio 21 Institute, University of Melbourne, Parkville VIC 3052, Australia

^6^Berlin-Brandenburg Institute of Advanced Biodiversity Research (BBIB), Berlin, Germany

**Tab. S1.** Primers used in the present study


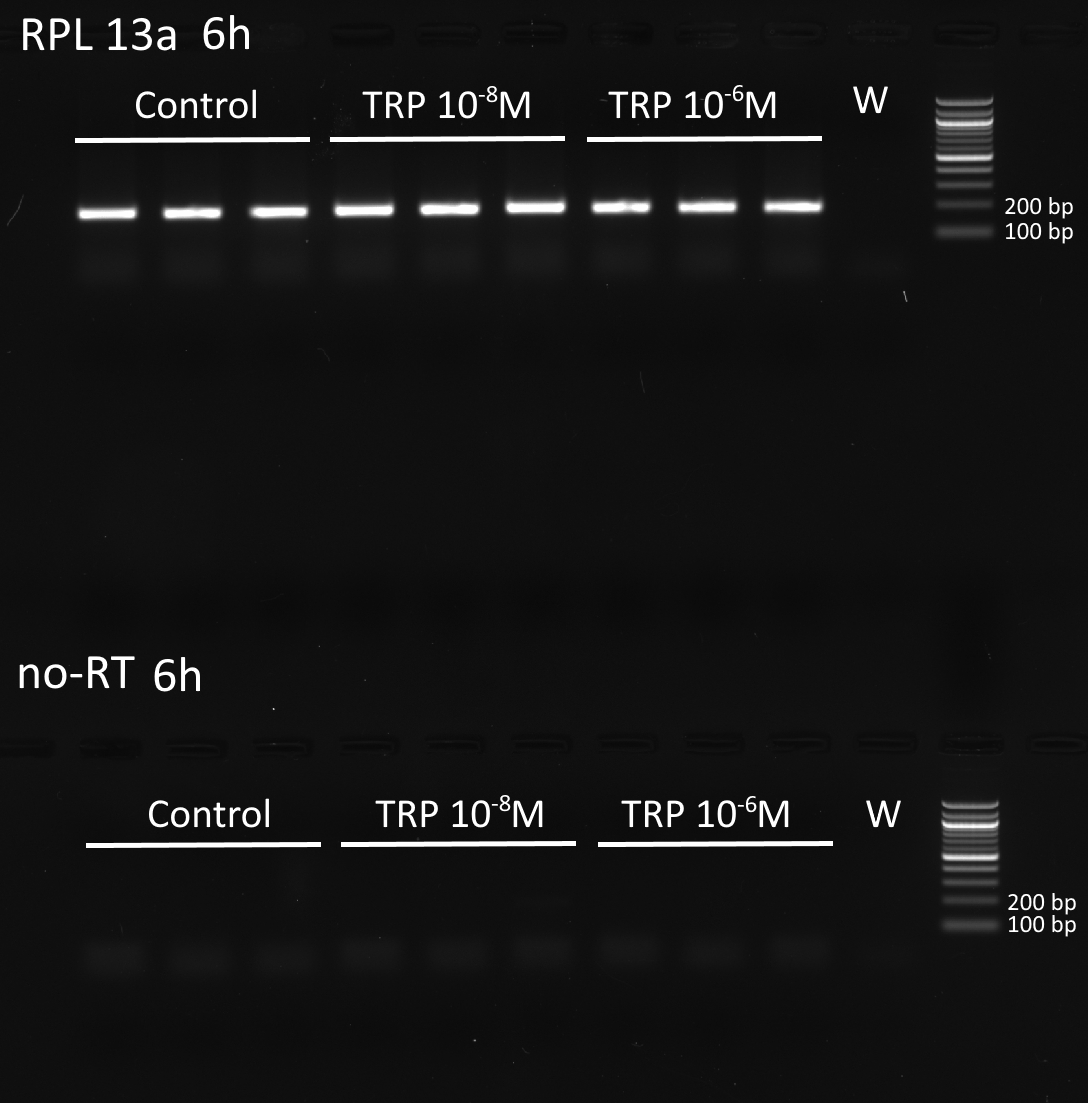

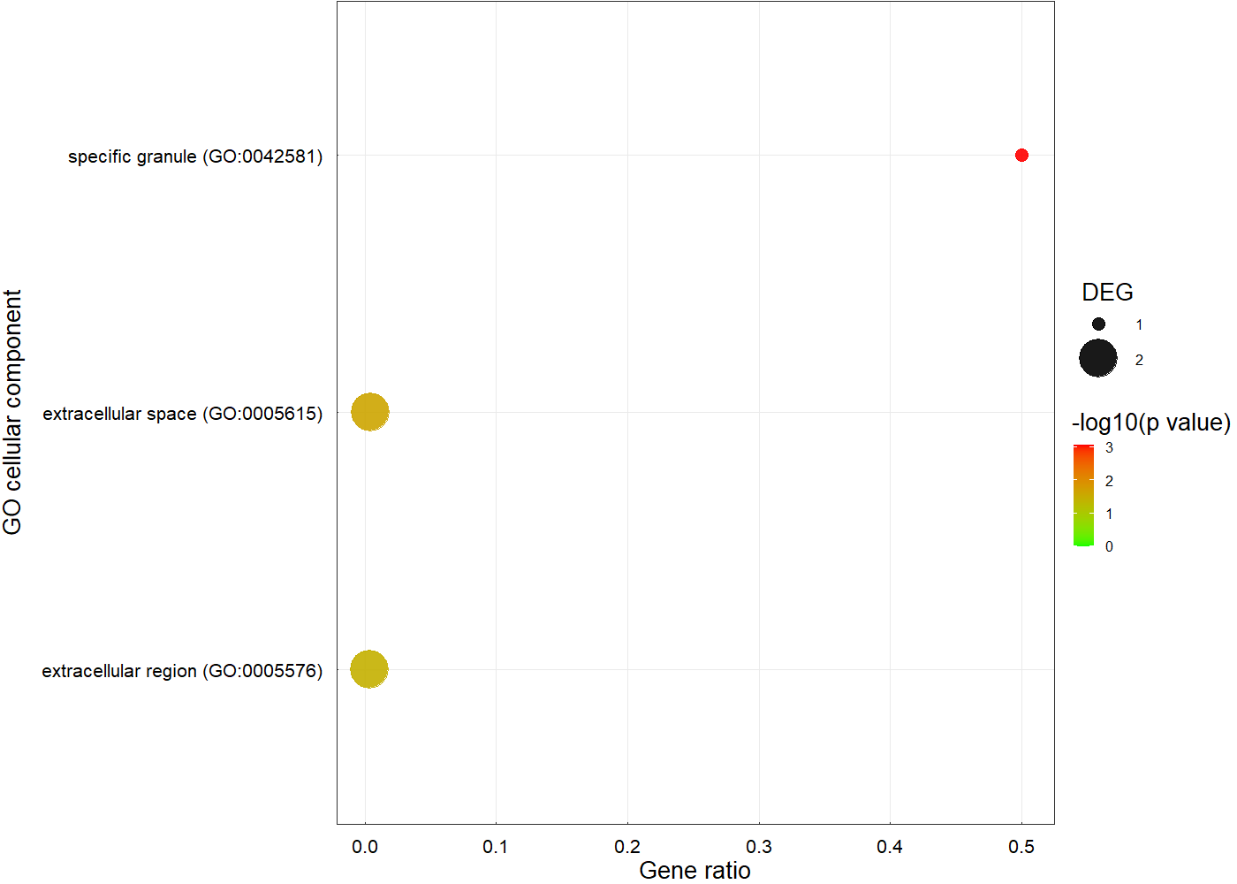


**Fig. S1.** Positive and negative control for Reverse transcription quantitative PCR (RT-qPCR) analysis. The samples were checked using Reverse transcription PCR (RT PCR) method. Electrophoresis of RT PCR products was performed using a 2.5% TAE agarose gel stained with ethidium bromide. RPL13a 6h - positive control of obtained samples; no-RT 6h – negative control without using of reverse transcriptase during transcription of RNA to cDNA; Control 6 – samples collected 6 h from individuals injected with physiological saline; TRP-7 10^-8^ and 10^-6^ 6h– samples collected 6 h from beetles injected with a solution of physiological saline and Tenmo-TRP-7 at a concentration of 10^-8^ and 10^-6^; W – water.

**
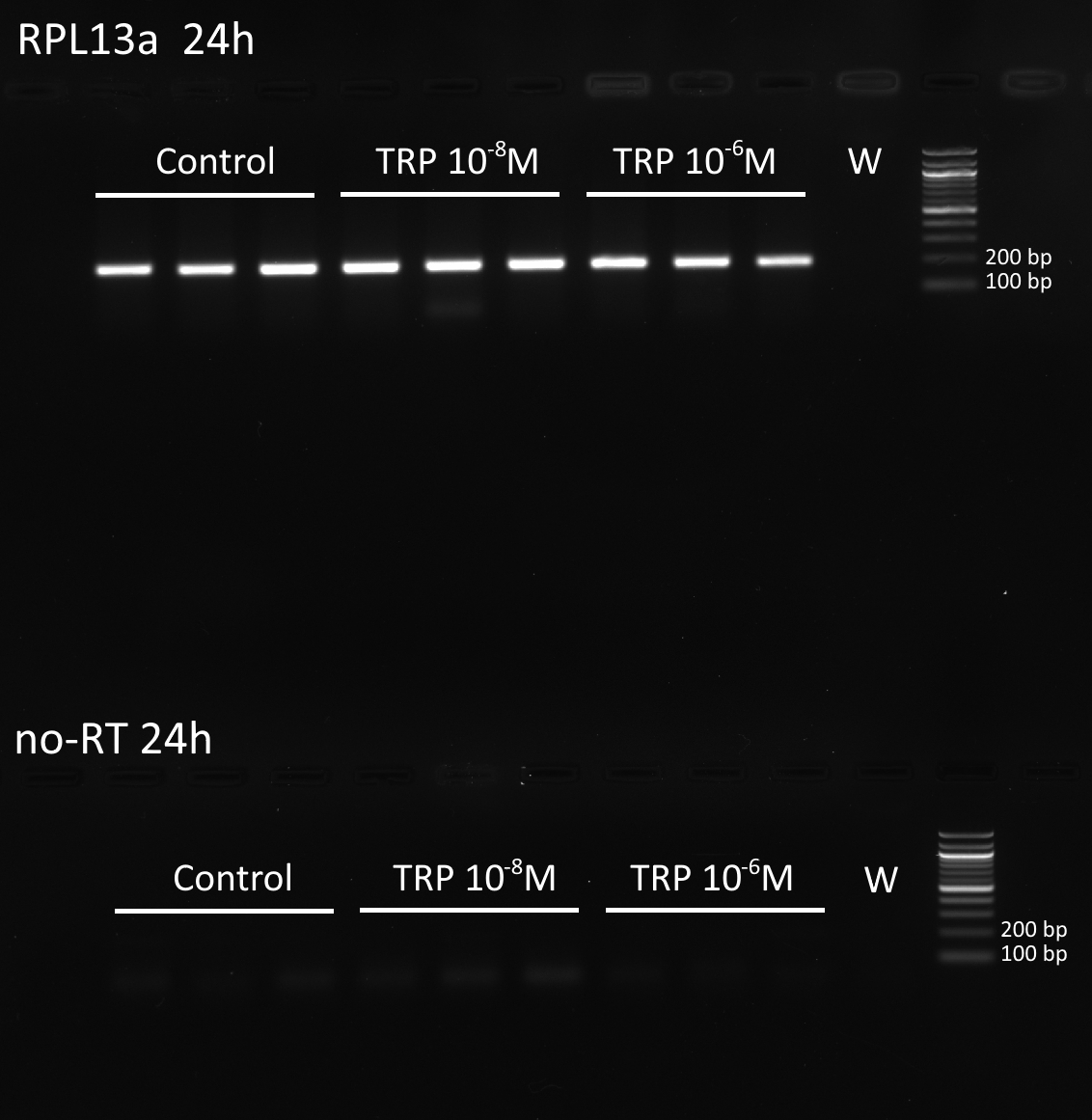
**

**Fig. S2.** Positive and negative control for Reverse transcription quantitative PCR (RT-qPCR) analysis. The samples were checked using Reverse transcription PCR (RT PCR) method. Electrophoresis of RT PCR products was performed using a 2.5% TAE agarose gel stained with ethidium bromide. RPL13a 24h - positive control of obtained samples; no-RT 24h – negative control without using of reverse transcriptase during transcription of RNA to cDNA; Control – samples collected 24 h from individuals injected with physiological saline; TRP-7 10^-8^ and 10^-6^– samples collected 24 h from beetles injected with a solution of physiological saline and Tenmo-TRP-7 at a concentration of 10^-8^ and 10^-6^; W – water.


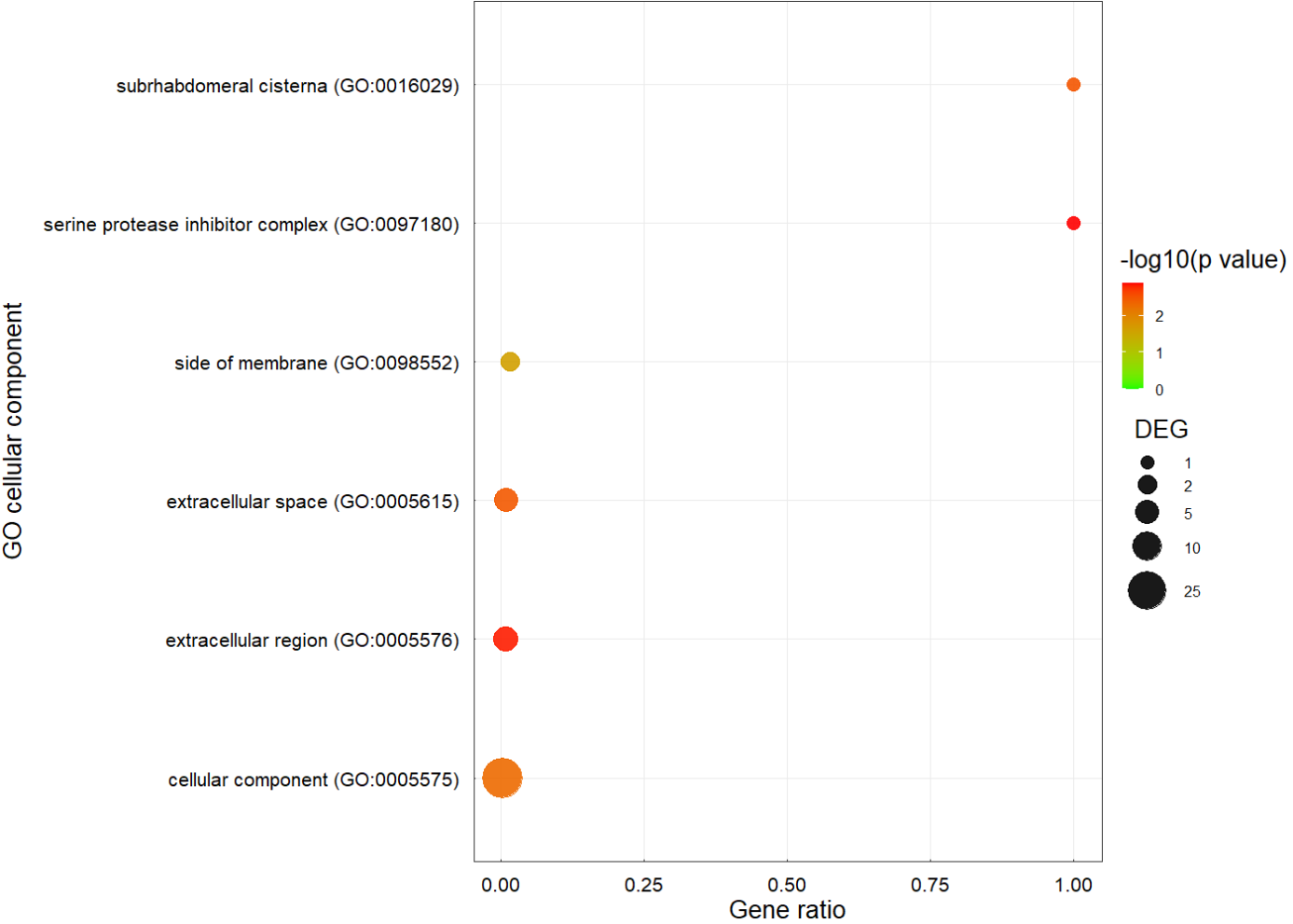
**Fig. S3.** GO enrichment analysis which present comparison of transcriptomic data between Control individuals and 6 hours after Tenmo-TRP-7 treatment at concentration of 10^-8^ M. The identification of the most representative GO terms of cellular component using REVIGO (http://revigo.irb.hr/) was performed (cut off <0.05). The size of the dots represents the number of genes in the significant differently expressed genes (DEGs). Gene ratio' is the percentage of total DEGs in the given GO term. For the graphical presentation of the obtained data, *ggplot2* (https://ggplot2.tidyverse.org) for RStudio was used (http://www.rstudio.com).


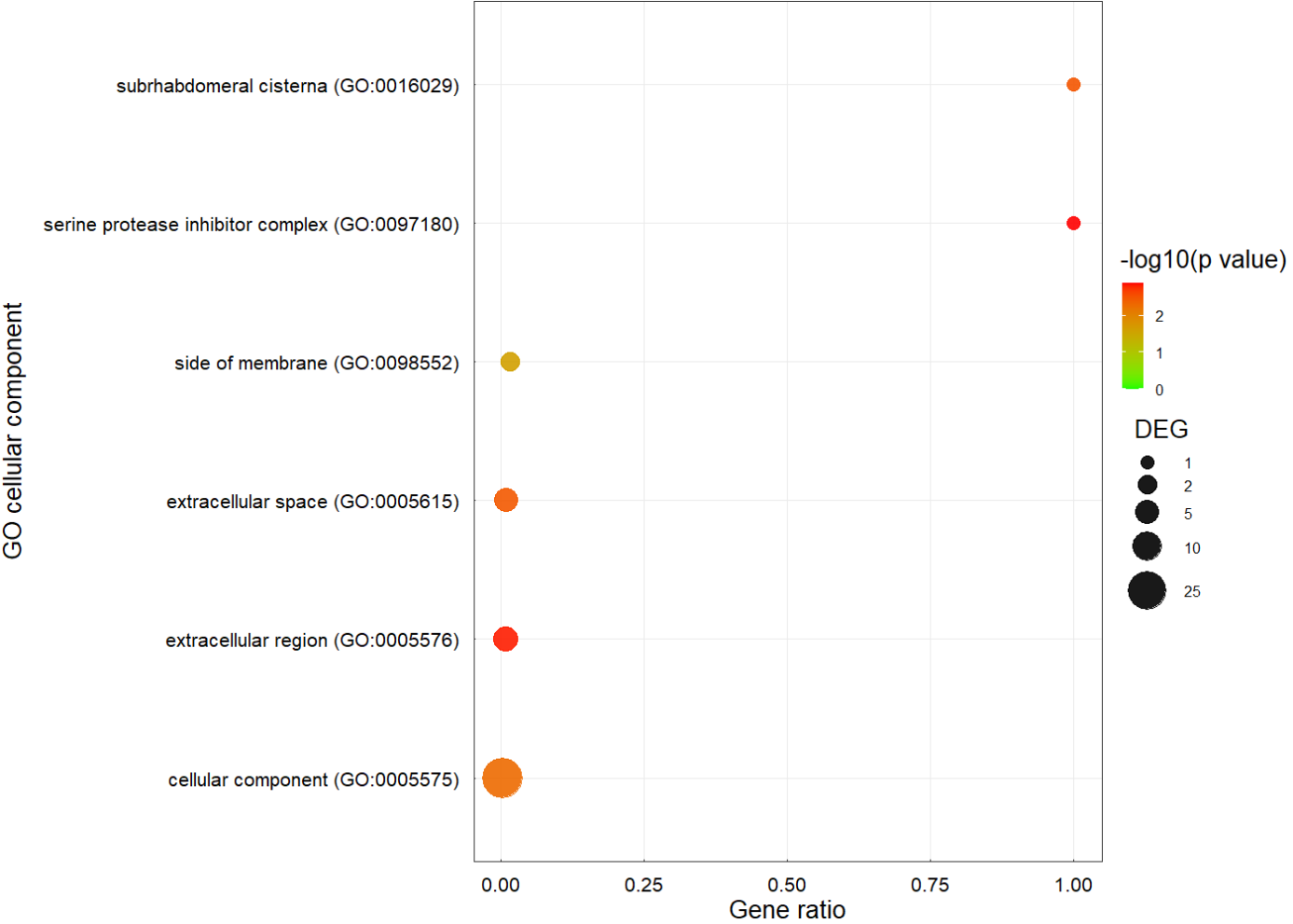


**Fig. S4.** GO enrichment analysis which present comparison of transcriptomic data between Control individuals and 6 hours after Tenmo-TRP-7 treatment at concentration of 10^-6^ M. The identification of the most representative GO terms of cellular component using REVIGO (http://revigo.irb.hr/) was performed (cut off <0.05). The size of the dots represents the number of genes in the significant differently expressed genes (DEGs). Gene ratio' is the percentage of total DEGs in the given GO term. For the graphical presentation of the obtained data, *ggplot2* (https://ggplot2.tidyverse.org) for RStudio was used (http://www.rstudio.com).


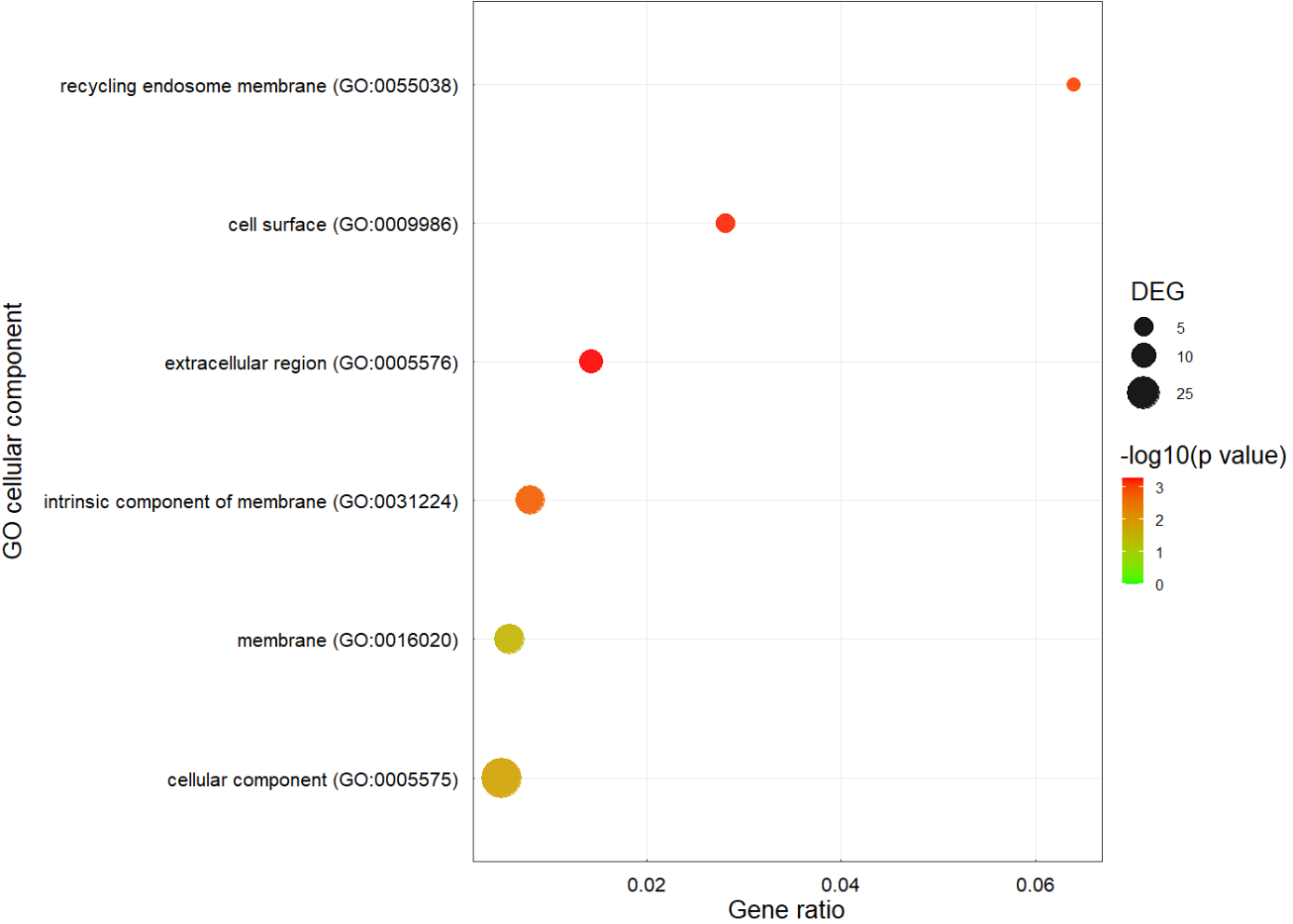


**Fig. S5.** GO enrichment analysis which present comparison of transcriptomic data between Control individuals and 24 hours after Tenmo-TRP-7 treatment at concentration of 10^-8^ M. The identification of the most representative GO terms of cellular component using REVIGO (http://revigo.irb.hr/) was performed (cut off <0.05). The size of the dots represents the number of genes in the significant differently expressed genes (DEGs). Gene ratio' is the percentage of total DEGs in the given GO term. For the graphical presentation of the obtained data, *ggplot2* (https://ggplot2.tidyverse.org) for RStudio was used (http://www.rstudio.com).


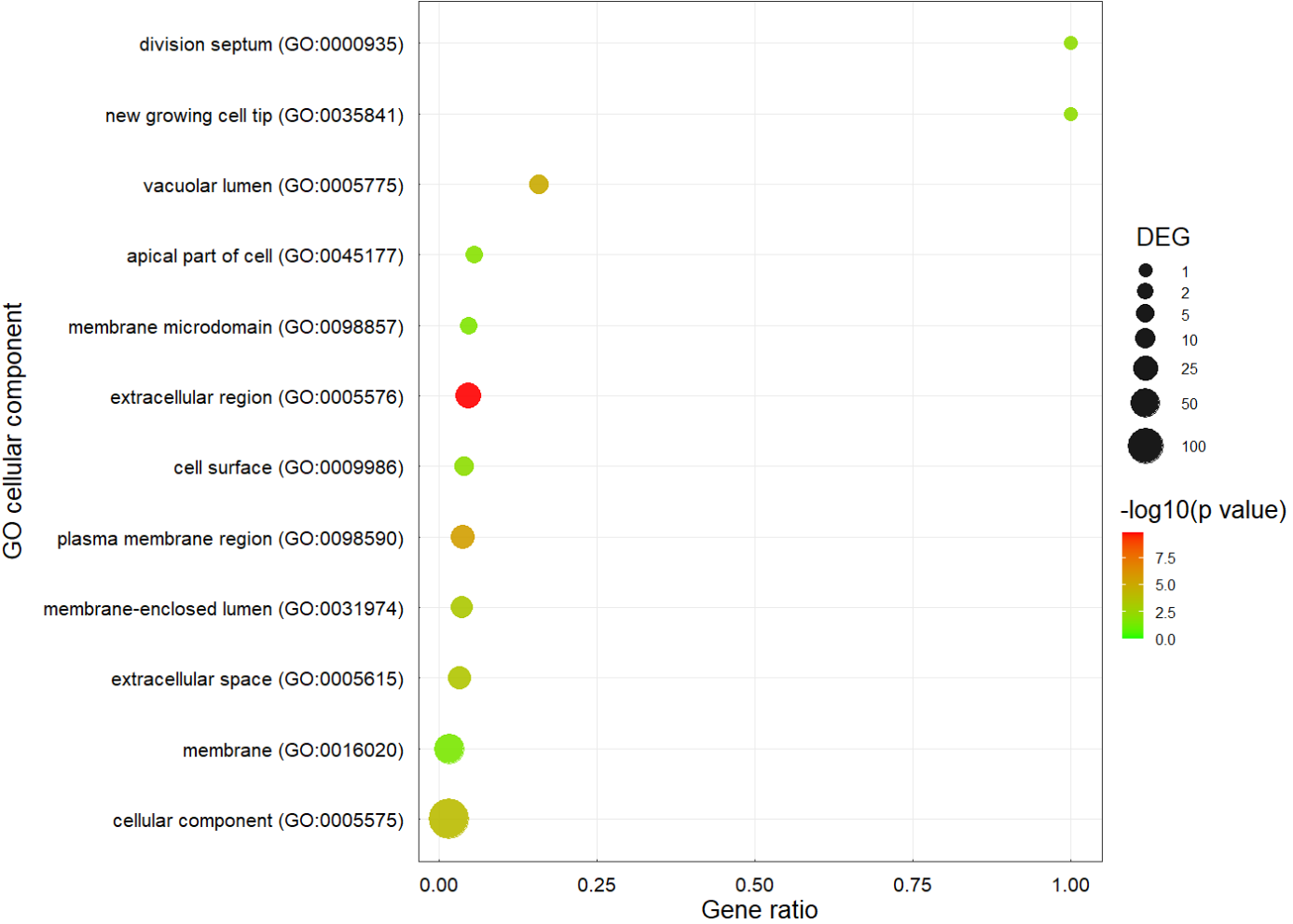


**Fig. S6.** GO enrichment analysis which present comparison of transcriptomic data between Control individuals and 24 hours after Tenmo-TRP-7 treatment at concentration of 10^-6^ M. The identification of the most representative GO terms of cellular component using REVIGO (http://revigo.irb.hr/) was performed (cut off <0.05). The size of the dots represents the number of genes in the significant differently expressed genes (DEGs). Gene ratio' is the percentage of total DEGs in the given GO term. For the graphical presentation of the obtained data, *ggplot2* (https://ggplot2.tidyverse.org) for RStudio was used (http://www.rstudio.com).
